# Chronic exposure to intestinal parasites and bacterial enteropathogens among children in rural Madagascar: Implications for asymptomatic carriage and co-infections

**DOI:** 10.1371/journal.pntd.0014519

**Published:** 2026-07-07

**Authors:** Wanesa Wilczyńska, Daniel Kasprowicz, Krzysztof Korzeniewski

**Affiliations:** 1 Department of Epidemiology and Tropical Medicine, Military Institute of Medicine -National Research Institute, Warsaw, Poland; 2 Clinique Médicale Beyzym, Manerinerina, Madagascar; University of North Carolina at Chapel Hill, UNITED STATES OF AMERICA

## Abstract

**Background:**

Intestinal infections remain highly prevalent among children living in rural, resource-limited settings, where repeated exposure to environmental pathogens begins early in life. In such contexts, enteric infections often represent chronic colonization rather than acute disease, frequently involving simultaneous carriage of parasitic and bacterial pathogens. Despite growing interest in intestinal co-infections, data on parasitic–bacterial co-occurrence, particularly among asymptomatic children in endemic settings, remain limited. Madagascar, characterized by high endemicity of intestinal parasites and major gaps in water and sanitation infrastructure, provides a relevant setting to investigate these patterns.

**Methodology/principal findings:**

A community-based cross-sectional study was conducted among 242 children under 15 years of age from three rural communities in northwestern Madagascar. Stool samples were examined using light microscopy for intestinal parasites and real-time PCR for selected protozoan and bacterial enteropathogens. At least one intestinal pathogen was detected in 64.9% of children. Parasitic infections predominated (46.7%), with *Giardia intestinalis* identified as the most common pathogen (43.4%), while bacterial enteropathogens were detected in 23.1% of participants, mainly *Campylobacter* spp. and diarrheagenic *Escherichia coli*. Mixed infections were frequent: 23.6% of children harbored two or more pathogens, and parasitic–bacterial co-infections accounted for a substantial proportion of detected cases. Notably, most infections—including mixed infections—were asymptomatic. Infection patterns varied across study sites and were associated with environmental exposures such as water source, sanitation access, contact with livestock, and exposure to surface water.

**Conclusions/significance:**

Intestinal parasitic–bacterial co-infections are common among children in rural Madagascar and often occur without overt clinical symptoms. These findings highlight the limitations of symptom-based and single-pathogen diagnostic approaches in endemic settings. Integrated diagnostic strategies combining microscopy and molecular methods, together with improvements in water and sanitation infrastructure, are essential to address the hidden burden of chronic intestinal infections in vulnerable pediatric populations.

## Author summary

Children living in rural, low-resource settings are exposed to intestinal microbes from a very early age. Many of these infections do not cause obvious symptoms, yet they may still affect child health, growth, and development. In this study, we examined intestinal infections among children from three rural communities in northwestern Madagascar, a region where access to clean water and sanitation is limited.

We found that intestinal infections were very common, particularly infections caused by parasites. Many children carried more than one pathogen at the same time, including combinations of parasites and bacteria. Importantly, most of these children did not show signs of illness, meaning that infections would likely remain unnoticed without laboratory testing. The types of infections and their frequency differed between communities and were linked to daily environmental exposures, such as water sources, sanitation conditions, and contact with animals.

Our findings show that focusing only on children with symptoms underestimates the true burden of intestinal infections in endemic settings. We also demonstrate that combining traditional microscopy with modern molecular methods provides a more complete picture of intestinal health. This work highlights the need for integrated diagnostic approaches and long-term improvements in water and sanitation to protect children living in vulnerable communities.

## Introduction

Gastrointestinal infections remain one of the most significant public health challenges in low- and middle-income countries, particularly among children living in rural areas [1,2]. In regions such as sub-Saharan Africa, limited access to safe drinking water, adequate sanitation, and basic healthcare services leads to continuous exposure to a wide range of intestinal pathogens from early childhood. As a result, intestinal infections in children often do not occur as isolated, acute disease episodes but rather represent a chronic process characterized by repeated colonization and reinfection, frequently without overt clinical symptoms [3,4].

In settings with a high endemicity of intestinal parasites, the presence of protozoa and helminths is often perceived as part of the biological “background” of the pediatric gut. At the same time, enteropathogenic bacteria, including various pathotypes of *Escherichia coli*, *Campylobacter* spp., and *Salmonella* spp., may periodically overlap with existing parasitic infections, resulting in complex patterns of pathogen co-occurrence [5–8]. Increasing evidence indicates that such chronic exposure and simultaneous carriage of multiple microorganisms do not necessarily correlate with clinical symptoms but may nevertheless affect nutritional status, intestinal barrier function, and the development of chronic low-grade inflammation [9,10].

Traditional approaches to intestinal infections, largely focused on the etiology of acute diarrhea, do not fully capture the epidemiological complexity observed in resource-limited settings. The development of molecular diagnostic methods, including multiplex real-time polymerase chain reaction assays, has enabled the simultaneous detection of a broad spectrum of intestinal pathogens with high sensitivity and specificity, even in asymptomatic individuals. The application of these techniques has revealed that a positive diagnostic result does not always indicate disease but often reflects asymptomatic carriage or chronic colonization, particularly among children with sustained exposure to environmental pathogens [11,12].

In this context, intestinal co-infections are of particular importance, understood not only as the concurrent presence of multiple pathogens but also as dynamic interactions between parasites and bacteria within the gastrointestinal tract. Such co-occurrence may modulate host immune responses, influence pathogen shedding intensity, and modify the risk of developing clinical symptoms [13,14]. Despite growing interest in this topic, data on chronic exposure to intestinal pathogens and patterns of parasitic–bacterial co-infections among children living in rural areas of sub-Saharan Africa remain limited, especially for asymptomatic populations.

Madagascar, characterized by a high prevalence of intestinal parasites and substantial deficiencies in sanitation infrastructure in rural regions, represents a model setting conducive to chronic intestinal pathogen carriage [15–17]. Understanding the frequency and patterns of parasitic–bacterial co-infections in this population is essential not only for the interpretation of diagnostic findings but also for the design of effective public health interventions aimed at improving sanitation and interrupting cycles of reinfection.

The aim of this study was to determine the prevalence of selected bacterial and parasitic intestinal pathogens among children under 15 years of age living in rural areas of northern Madagascar. We further aimed to assess whether intestinal pathogen carriage is associated with (i) environmental exposure factors, (ii) asymptomatic infection status and co-infections, and (iii) selected haematological parameters as markers of systemic response.

This assumption is supported by evidence indicating that even asymptomatic intestinal infections may be associated with chronic low-grade inflammation, modulation of host immune responses, and nutritional deficiencies, which may be reflected in peripheral blood parameters such as leukocyte profiles and hemoglobin levels [9,10,18]. We hypothesised that intestinal pathogen carriage is associated with environmental exposure factors and that infected children may show altered haematological parameters reflecting systemic inflammatory response.

## Materials and methods

### Ethics statement

The study received formal approval from the Service de Santé Publique du District d’Ambatoboeny, Ministry of Public Health, Madagascar (Approval No. 24/04 AB 2024). The study was conducted in accordance with the principles of the World Medical Association Declaration of Helsinki. Participation was entirely voluntary, and written informed consent for each child was obtained from a parent or legal guardian. Personal and biological data were handled confidentially and used exclusively for research purposes.

### Study design and study area

**Fig 1:**
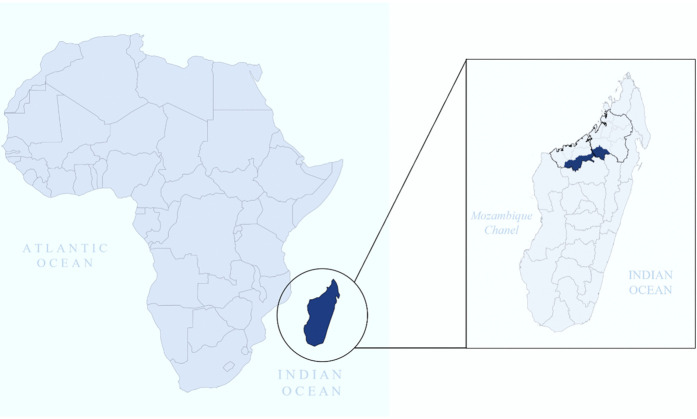
Study area. The location of Madagascar within the African continent is shown on the left. Thick black lines delineate the Boeny and Sofia regions, while the Ambato-Boina and Mampikony districts are highlighted in dark blue.

This study was designed as a cross-sectional, community-based survey with random participant selection. Screening for intestinal infections was conducted in August 2025 (within a single field campaign), during the dry season, in three rural communities in northwestern Madagascar: Manerinerina (Ambatoboeny District, Boeny Region), Bekoratsaka (Mampikony District, Sofia Region), and Antanadava (Mampikony District, Sofia Region) (Fig 1).

The map was created in R software using administrative boundary shapefiles from Natural Earth (Admin 0 – Countries and Admin 1 – States/Provinces, 1:10m cultural vectors). Source: https://www.naturalearthdata.com/downloads/10m-cultural-vectors/. Terms of use: https://www.naturalearthdata.com/about/terms-of-use/ (public domain).

The studied communities are characterized by livelihoods based predominantly on subsistence agriculture, with a particular focus on rice and groundnut cultivation and cattle husbandry. Access to sanitation infrastructure, safe drinking water, and healthcare services is limited in these settings, which facilitates the persistence of endemic intestinal infections and is consistent with conditions reported in other rural regions of Madagascar.

Base maps were generated using R software, and figure annotations were added using presentation software.

### Study participants: Inclusion and exclusion criteria

Children under 15 years of age residing in the study areas were eligible for inclusion if their parents or legal guardians provided written informed consent and completed an epidemiological questionnaire. Both children presenting with gastrointestinal symptoms and asymptomatic children were included, regardless of recent use of antimicrobial agents.

Exclusion criteria comprised age ≥15 years, lack of consent to participate, failure to complete the epidemiological questionnaire, or failure to provide a stool sample for analysis. Participation in the study was entirely voluntary. Of the 246 registered participants, four children were excluded from the final analysis: three due to incomplete questionnaires and one due to a mismatch between the biological sample identification number and questionnaire data. No caregivers declined participation. Ultimately, 242 children were included in the analysis.

### Collection of epidemiological and clinical data

Epidemiological, clinical, and socio-economic data were collected using a structured questionnaire translated into Malagasy. Questionnaires were administered with the assistance of trained medical staff from Clinique Médicale Beyzym in Manerinerina, who provided clarification to caregivers when needed.

The questionnaire included socio-demographic data (age, sex, place of residence), health-related information (symptoms potentially associated with intestinal infections, hospitalizations, medication use), and environmental, cultural, and socio-economic factors. Gastrointestinal and general symptoms included fever, abdominal pain, diarrhea, constipation, skin rash or itching, and other complaints such as vomiting. Antibiotic and/or antiparasitic treatment was assessed for the 28 days preceding enrollment, while hospitalizations were recorded for the three months prior to inclusion. Participation in deworming programs was classified as regular, irregular, or absent. Sanitation access was defined as use of a flush toilet, latrine, or lack of sanitation facilities. For analytical purposes, sanitation was further classified as “improved” or “unimproved” according to WHO/UNICEF Joint Monitoring Programme criteria. Improved sanitation included facilities that hygienically separate human excreta from human contact (e.g., flush toilets and improved latrines), whereas unimproved sanitation comprised shared, rudimentary, or absent sanitation facilities. Drinking water sources, contact with livestock and exposure to surface water were also recorded.

### Study procedures and biological sample collection

Participant registration was conducted by trained staff at Clinique Médicale Beyzym in Manerinerina. Following written informed consent and questionnaire completion, each participant was assigned a unique identification number used for both questionnaire documentation and biological samples. Venous blood samples were collected from each child and analyzed on site to determine hematological profiles using an automated hematology analyzer (Mindray BC-3000 Plus). Caregivers were then provided with a sterile stool container and verbal instructions for sample collection.

For logistical reasons, a single stool sample was collected from each participant, which was acknowledged as a limitation of the study. Upon receipt, stool samples were divided: one portion was applied to Whatman 903 protein saver cards (Whatman International, Maidstone, UK), air-dried at room temperature, labeled with the participant identification number, and stored in zip-lock bags with desiccant; the remaining portion was preserved in sodium acetate–acetic acid–formalin (SAF) solution.

Stool samples and filter paper cards were subsequently transported to Poland for further diagnostic analyses.

### Parasitological diagnostics – light microscopy

Stool samples and filter paper cards were transported to the Department of Epidemiology and Tropical Medicine, Military Institute of Medicine – National Research Institute (Gdynia, Poland), where further laboratory analyses were performed. Stool samples were used for microscopic diagnosis of intestinal parasites, while filter paper cards were reserved for molecular analyses.

Microscopic examination was performed as the first diagnostic step. Each stool sample was analyzed using three complementary parasitological methods: direct smear in Lugol’s iodine, Fulleborn flotation using saturated sodium chloride solution, and sedimentation by decantation in distilled water [19]. Direct smears and sedimentation preparations were examined at 10× and 40× magnification, whereas flotation preparations were examined at 10× magnification. All preparations were assessed for the presence of developmental stages of intestinal parasites, including cysts, eggs, and larvae.

The use of multiple microscopic techniques aimed to increase detection sensitivity for parasites with different densities and morphological characteristics, while accounting for limitations related to single-sample analysis.

### Molecular diagnostics – real-time PCR

Filter paper cards containing stool material were used for nucleic acid extraction. One complete filter spot was excised from each card using scissors and placed into a sterile 2 mL microcentrifuge tube. To minimize the risk of cross-contamination, scissors were decontaminated with a hypochlorite solution and rinsed with distilled water after each use.

Excised filter paper fragments were incubated in distilled water at room temperature for 15–20 hours to elute biological material. Following incubation, 400 µL of eluate was collected and mixed with LTX buffer from the Bosphore Nucleic Acid Extraction Versatile Spin Kit (Anatolia Geneworks, Istanbul, Turkey) and 20 µL of proteinase K. Samples were incubated at 56°C for 2 hours. Nucleic acid extraction was performed according to the manufacturer’s instructions using the protocol for stool samples. The final elution volume was 150 µL. Extracted nucleic acids were aliquoted and stored at −20°C until further molecular analyses.

Extracted nucleic acids were used for the detection of selected intestinal protozoa using real-time polymerase chain reaction assays. Molecular analyses were performed at the Department of Epidemiology and Tropical Medicine (Gdynia, Poland) using the AriaMx Real-Time PCR System (Agilent Technologies, USA).

Real-time PCR assays targeting *Cyclospora cayetanensis, Giardia intestinalis, Entamoeba histolytica, Cryptosporidium* spp., *Enterocytozoon bieneusi, Encephalitozoon* spp., and *Cystoisospora belli* were conducted using pathogen-specific primers and probes. Primer and probe sequences, fluorophores, quenchers, and corresponding references are provided in Table 1. Assays for *C. cayetanensis*, *G. intestinalis*, *E. histolytica*, and *C. belli* were performed in singleplex format, whereas multiplex assays were used for microsporidia (*E. bieneusi* and *Encephalitozoon* spp.) and *Cryptosporidium* spp.

**Table 1:** Primers and probes used in this study.

Pathogen		Sequences (5′ → 3′)	Gene	Reference
***G. intestinalis***	F	GACGGCTCAGGACAACGGTT	18S rRNA	[20]
	R	TTGCCAGCGGTGTCCG		
	P	FAM-CCCGCGGCGGTCCCTGCTAG-BHQ1		
***E. histolytica***	F	GTTTGTATTAGTACAAAATGGCCAATTC	18S rRNA	[21]
	R	TCGTGGCATCCTAACTCACTTAGA		
	P	FAM-CAATGAATTGAGAAATGACA-MGB		
***C. cayetanensis***	F	TAGTAACCGAACGGATCGCATT	18S rRNA	[22]
	R	AATGCCACGTAGGCCAATA		
	P	FAM-CCGGCGATAGATCATTCAAGTTTCTGACC-TAMRA		
***C. belli***	F	ATATTCCCTGCAGCATGTCTGTTT	ITS-2	[23]
	R	CCACACGCGTATTCCAGAGA		
	P	FAM-CAAGTTCTGCTCACGCGCTTCTGG-BHQ1		
***Cryptosporidium* spp.**	F	CATGGATAACCGTGGTAAT	18S rRNA	[24]
	R	TACCCTACCGTCTAAAGCTG		
	P	FAM-CTAGAGCTAATACATGCGAAAAAA-MGB-BHQ1		
***E. bieneusi***	F	CGCTGTAGTTCCTGCAGTAAACTATGCC	18S rRNA	
	R	CTTGCGAGCGTACTATCCCCAGAG		
	P	HEX- ACGTGGGCGGGAGAAATCTTAGTGTTCGGG -BHQ1		
***Encephalitozoon* spp.**	F	GCAAGGGAGGAATGGAACAGAACAG	18S rRNA	
	R	CACGTTCAGAAGCCCATTACACAGC		
	P	TxRd-CGGGCGGCACGCGCACTACGATA-BHQ2		

F – forward primer; R – reverse primer; P – probe; BHQ1 - black hole quencher; MGB - minor groove binding; TxRd – Texas Red; FAM, HEX, TxRd - fluorescent dye

Samples positive for *E. histolytica* were further tested using the commercial AmpliTest *Entamoeba histolytica* kit (Amplicon, Wrocław, Poland) to differentiate pathogenic *E. histolytica* from non-pathogenic *E. dispar*. Primers and probes were supplied as lyophilized reagents and reconstituted according to the manufacturer’s instructions. Working solutions were prepared and stored at −20°C to minimize freeze–thaw cycles.

Real-time PCR reactions were performed using HS-PCR Mix Probe (A&A Biotechnology, Poland). Amplification conditions were optimized individually for each primer–probe set based on melting temperatures. Thermal cycling conditions included an initial denaturation at 95°C for 5 minutes, followed by 45 cycles of denaturation at 95°C for 15 seconds and annealing/extension at assay-specific temperatures for 45 seconds.

Reactions were performed in single runs, as molecular diagnostics were intended to complement microscopic examination. Samples yielding ambiguous or borderline results were reanalyzed. A cycle threshold value <38 was considered positive. The cut-off was defined based on the total number of amplification cycles (45 cycles) and empirical observation of amplification curves, where signals above cycle 38 showed inconsistent fluorescence kinetics and loss of exponential amplification phase. Given the use of filter paper–based stool eluates and the absence of external quantitative standards for this matrix, a conservative threshold was applied to minimise the risk of false-positive results associated with late non-specific amplification. Each run included a positive control consisting of stool samples previously confirmed as positive by microscopy (in-house reference material) and a negative control (distilled water). No internal inhibition control was used for protozoan assays.

Bacterial enteropathogens were detected using the Bosphore Bacterial GI Panel Kit v1 (Anatolia Geneworks, Istanbul, Turkey), designed for real-time PCR detection of selected bacterial causes of gastrointestinal infections in stool samples. The panel simultaneously detects tcdA/tcdB genes of *Clostridium difficile* (toxins A/B), 16S rRNA gene of *Campylobacter* spp. (*C. jejuni, C. upsaliensis, C. coli, C. lari*), ttrB gene of *Salmonella* spp., ipaH gene of enteroinvasive *Escherichia coli*/ *Shigella* spp., stx-1/stx-2 genes of Shiga toxin–producing *E. coli* (stx1/stx2), and ail gene of *Yersinia enterocolitica*. Analyses were conducted according to the manufacturer’s instructions using two reaction mixtures. The kit includes an integrated internal control to monitor DNA extraction efficiency, PCR performance, and potential amplification inhibition. Each run also included a positive control (synthetic DNAs) and a negative control (distilled water). Amplification and fluorescence detection were performed using the AriaMx Real-Time PCR System, and results were interpreted according to the manufacturer’s criteria. Real-time PCR amplification was performed under the following cycling conditions: initial denaturation at 95°C for 5 min, followed by 40 cycles of denaturation at 97°C for 15 s, annealing/extension at 60°C for 60 s (with fluorescence data acquisition at this step), and a final hold step at 32°C for 20 s. As with protozoan diagnostics, analyses were performed once, with repeat testing for borderline results and a cycle threshold value <32 was considered positive. This threshold was aligned with the manufacturer’s performance specifications for the internal control (IC), for which Ct values above 32 indicate suboptimal amplification or potential inhibition. In our assay conditions (40 cycles), this cut-off ensured that only results within the validated dynamic range of the assay were considered positive, with the positive control consistently amplifying at Ct ~ 28, confirming expected assay performance.

For commercial kits (AmpliTest and Bosphore Bacterial GI Panel), the exact oligonucleotide sequences are proprietary and were not disclosed by the manufacturers. To comply with MIQE guidelines, a detailed overview of the available assay characteristics, target genes, fluorophores, and validation profiles for these commercial platforms is provided in S1 Table (Supporting Information).

The selection of bacterial enteropathogens was based on their established epidemiological relevance as major causes of acute gastroenteritis in paediatric populations, as highlighted by the WHO in global burden assessments of diarrhoeal and foodborne diseases [25]. At the same time, in high-endemicity, resource-limited settings, these pathogens are frequently detected in asymptomatic individuals, reflecting colonization or continuous environmental exposure rather than clinically manifest infection [9].

### Statistical analysis

Epidemiological, clinical, and environmental data were entered into Microsoft Excel (Microsoft Corporation, Redmond, WA, USA) and subsequently processed and analyzed using R software (version 4.5.2; R Foundation for Statistical Computing, Vienna, Austria). Initial data exploration included calculation of frequencies and percentages for categorical variables, as well as means, medians, and standard deviations for continuous variables. The prevalence of individual infections and co-infections was expressed as the proportion of children with a detected pathogen relative to the total study population. Differences between infection groups for categorical variables (e.g., water source, sanitation access, contact with livestock) were assessed using Fisher’s exact test or the chi-square test, depending on cell counts. To estimate the strength of associations between environmental variables and infection status, binary logistic regression models were applied. Logistic regression was also used to analyze infection complexity (mono-infection versus co-infection) using an appropriate dependent variable. Regression results were reported as odds ratios (ORs) with 95% confidence intervals (CIs). Associations between infection type (single and mixed parasitic and bacterial infections) and the presence of gastrointestinal symptoms were evaluated using Pearson’s chi-square test. When statistically significant results were observed, Pearson residuals were examined to identify categories with over- or under-representation of symptomatic or asymptomatic cases. Hematological parameters, including white blood cell count, lymphocytes, granulocytes, hemoglobin, hematocrit, and platelet count, were compared between infection groups using the Kruskal–Wallis test for non-parametric data. For each infection group, means ± standard deviation (SD), 95% confidence intervals were calculated.

Given the exploratory nature of the analyses and the number of comparisons performed, no adjustment for multiple testing was applied; results should therefore be interpreted with caution.

All statistical tests were two-sided, and a p-value < 0.05 was considered statistically significant.

## Results

### Study population characteristics

A total of 242 children were included in the study. Socio-demographic and environmental exposure characteristics are presented in Table 2.

**Table 2:** Socio-demographic and environmental characteristics of the study population (n = 242), Madagascar 2025.

	*n*	%
**Sex**		
Male	121	50.0
Female	121	50.0
**Age group (years)**		
<5	93	38.4
5–10	39	16.1
>10	110	45.5
**Place of residence**		
Manerinerina	130	53.7
Bekoratsaka	86	35.6
Antanadava	26	10.7
**Gastrointestinal symptoms**		
Yes	68	28.1
No	174	71.9
**Water source**		
Improved sanitation	28	11.6
Well/river	214	88.4
**Sanitation**		
Flush toilet	12	5.0
Latrine	90	37.2
None	140	57.9
**Livestock contact**		
Yes	159	65.7
No	83	34.3
**Stagnant water exposure**		
Yes	164	67.8
No	78	32.2
**Deworming**		
Regular	12	5.0
Irregular	43	17.8
None	187	77.2
**Antibiotic use (last 3 months)**		
Yes	16	6.6
No	226	93.4

### Prevalence of intestinal pathogens

Among the 242 children included in the study, 113 (46.7%) were infected with at least one intestinal parasite. Helminths were detected in 14 children (5.8%), most commonly *Hymenolepis nana* (n = 8) and hookworm (n = 5), while *Trichuris trichiura* was identified in a single child. Helminth mono-infections were observed in three children, whereas 11 children had co-infections with helminths and other parasites.

The most prevalent protozoan pathogen was *G. intestinalis*, detected in 105 children (43.4%). Mono-infections occurred in 50 children, and 55 children had co-infections with other pathogens, including coccidia (n = 1), microsporidia (n = 4), helminths (n = 8), or non-pathogenic parasites (n = 42). Coccidia were found in three children (1.2%), including one child infected with two species (*C. belli* and *C. cayetanensis*). Microsporidia were detected in seven children (2.9%), mainly *Enterocytozoon* spp. (n = 6) and *Encephalitozoon* spp. (n = 1).

Among non-pathogenic parasites, *Blastocystis* spp. (n = 74; 30.6%), *Entamoeba coli* (n = 33; 13.6%), and *Endolimax nana* (n = 15; 6.2%) were the most frequent. Eight samples were initially suspected for *E. histolytica*, but species differentiation confirmed all as the non-pathogenic *E. dispar* (n = 8; 3.3%) (Tables 3, S2).

**Table 3:** Frequency of intestinal parasite and bacterial infections among children (n = 242) (monoinfections and coinfections), Madagascar 2025.

	Number of infected children (*n*)	Percentage of infected children (%)
**HELMINTHS**	**14**	**5.8**
**Hookworm**	5	2.1
***Hymenolepis nana***	8	3.3
***Trichuris trichiura***	1	0.4
monoinfections	3	1.2
co-infections	11	4.5
**PATHOGENIC PARASITES**	**116**	**47.9**
***Giardia intestinalis***	105	43.4
monoinfections	50	20.7
co-infections	55	22.7
**Coccidia**	4	1.7
monoinfections	0	0.0
co-infections	3	1.2
**Microsporidium**	7	2.9
monoinfections	3	1.2
co-infections	4	1.7
**NONPATHOGENIC PARASITES**	**132**	**54.5**
**BACTERIA**	**88**	**36.4**
***Salmonella* spp.**	0	0.0
***Yersinia enterocolitica***	0	0.0
***Clostridium difficile***	0	0.0
***Campylobacter* spp.**	**45**	**18.6**
monoinfections	9	3.7
co-infections	36	14.9
***E. coli* (VTEC)/(STEC)**	**17**	**7.0**
monoinfections	2	0.8
co-infections	15	6.2
***E. coli* (EIEC)/ *Shigella* spp**	**36**	**14.9**
monoinfections	10	4.1
co-infections	26	10.7

In this population*, G. intestinalis* was detected by light microscopy in 69 children (28.5%), whereas real-time PCR identified infections in 105 children (43.4%), corresponding to 36 additional cases not detected by microscopy. All microscopy-positive cases were confirmed by real-time PCR.

Among all children included in the study, 56 (23.1%) were infected with at least one bacterial gastrointestinal pathogen. Among pathogenic bacteria*, Campylobacter* spp. was detected in 45 children (18.6%), including 9 mono-infections and 36 co-infections, mainly with protozoan parasites. *E. coli* VTEC/STEC was detected in 17 children (7.0%) (2 mono-infections, 15 co-infections), while *E. coli* EIEC/ *Shigella* was identified in 36 children (14.9%) (10 mono-infections, 26 co-infections). *Salmonella* spp., *Y. enterocolitica,* and *C. difficile* were not detected in any sample. The prevalence of bacterial pathogens, including bacterial and bacterial–parasitic co-infections, is summarized in Tables 3 and S3.

**Fig 2:**
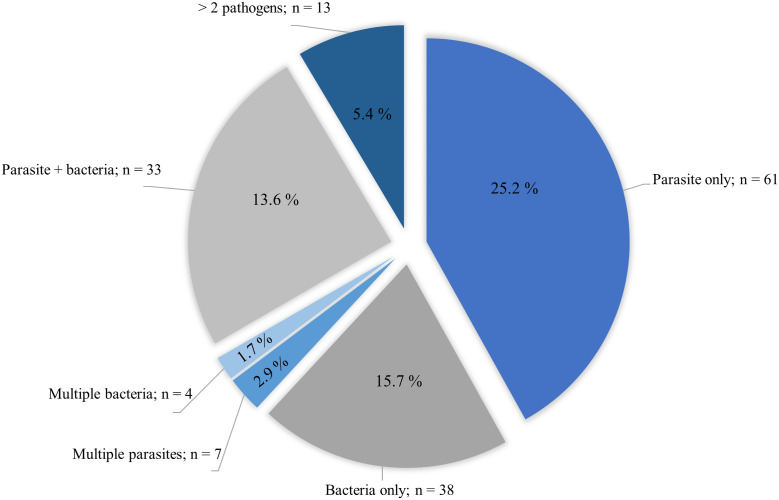
Distribution of intestinal pathogens among children (coinfection and monoinfection) (n = 242), Madagascar 2025.

Among the 242 children included in the study, 157 (64.9%) were infected with at least one intestinal pathogen. Co-infection analysis revealed complex infection patterns: 57 children (23.6%) harbored two or more intestinal pathogens (Fig 2). Multi-pathogen infection was defined as the detection of two or more distinct pathogen species in a single sample, irrespective of pathogen group (bacterial, protozoan, or mixed bacterial–protozoan co-detection).

### Demographic determinants

**Fig 3:**
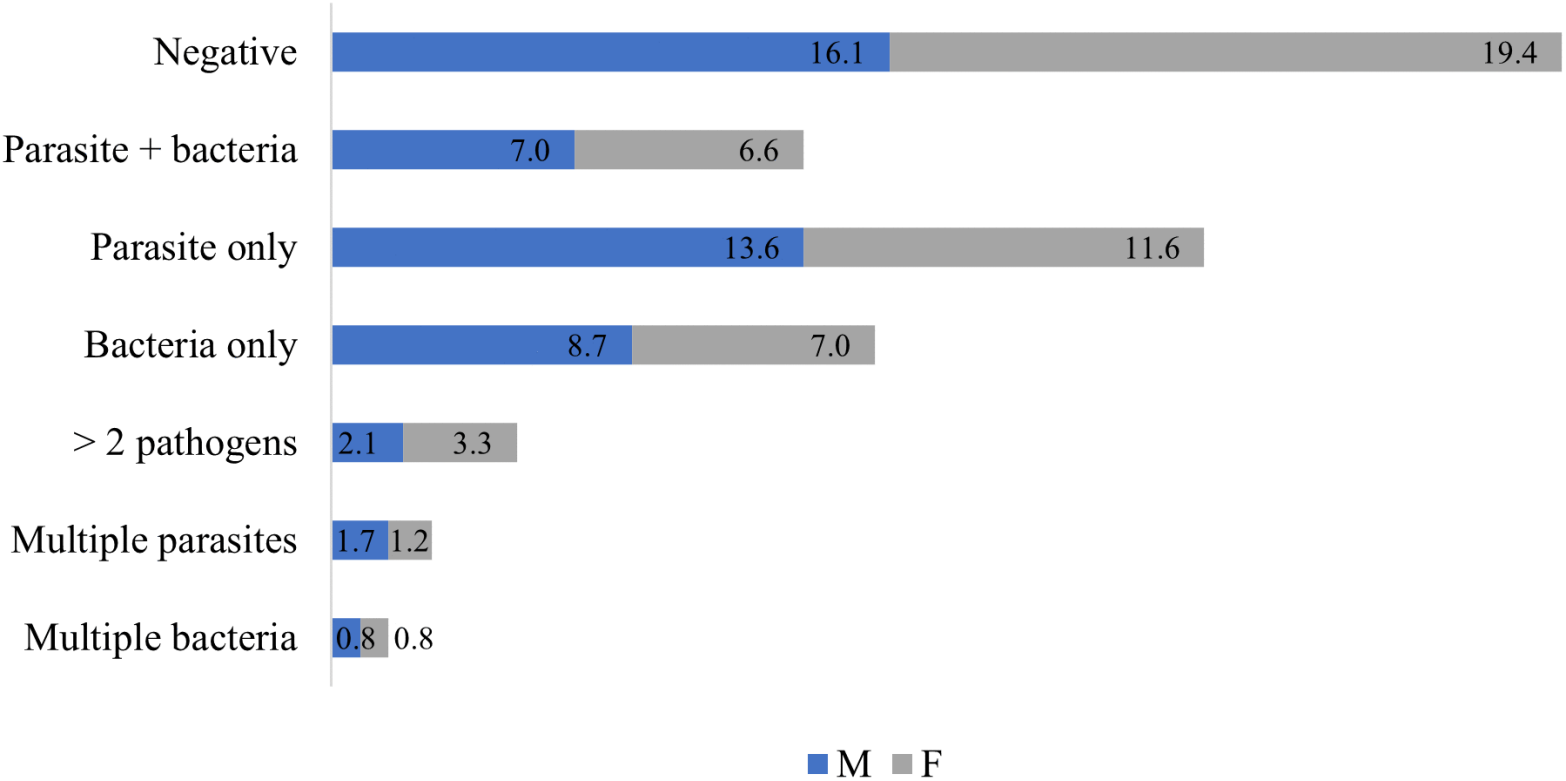
Intestinal infections among children by sex (n = 242), Madagascar 2025.

The study included 121 boys and 121 girls. The distribution of intestinal infections was similar between sexes, with no significant differences in the prevalence of mono-infections, co-infections, or negative results (Fig 3).

Age distribution was as follows: 38.4% of children were younger than 5 years, 45.5% were 5–10 years old, and 16.1% were older than 10 years. Infection patterns varied by age group, with the highest burden observed in children aged 5–10 years, who had the largest number of both mono-infections and co-infections.

Parasitic mono-infections were the most common infection type across all age groups, peaking in children aged 5–10 years (n = 37; 15.3% of the total population). Among children younger than 5 years, parasitic mono-infections were also frequent (n = 19; 7.9%), whereas children older than 10 years had a markedly lower prevalence (n = 5; 2.1%).

Bacterial mono-infections were more common in children aged 5–10 years (n = 17; 7.0%) than in younger (n = 13; 5.4%) or older children (n = 8; 3.3%). A similar pattern was observed for parasitic–bacterial co-infections, which were most frequent in children <5 years (n = 12; 5.0%) and 5–10 years (n = 17; 7.0%), and less common in children >10 years (n = 4; 1.7%).

**Fig 4:**
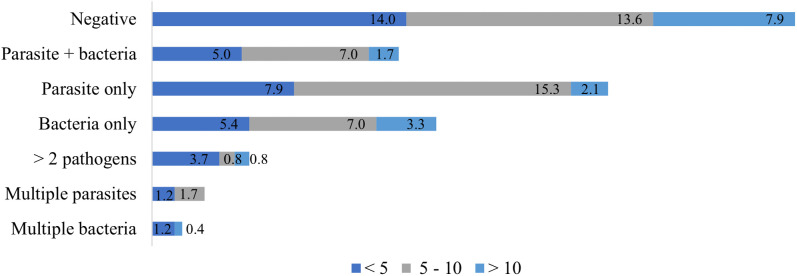
Intestinal infections among children by age (n = 242), Madagascar 2025.

Infections involving more than two pathogens (>2 pathogens) were mainly observed in children younger than 5 years (n = 9; 3.7%), whereas they were rare in older age groups (5–10 years: n = 2; 0.8%; > 10 years: n = 2; 0.8%). Similarly, multiple bacterial and multiple parasitic infections primarily affected younger children, with multiple parasitic infections not detected in children older than 10 years (Fig 4).

### Clinical correlates

**Fig 5:**
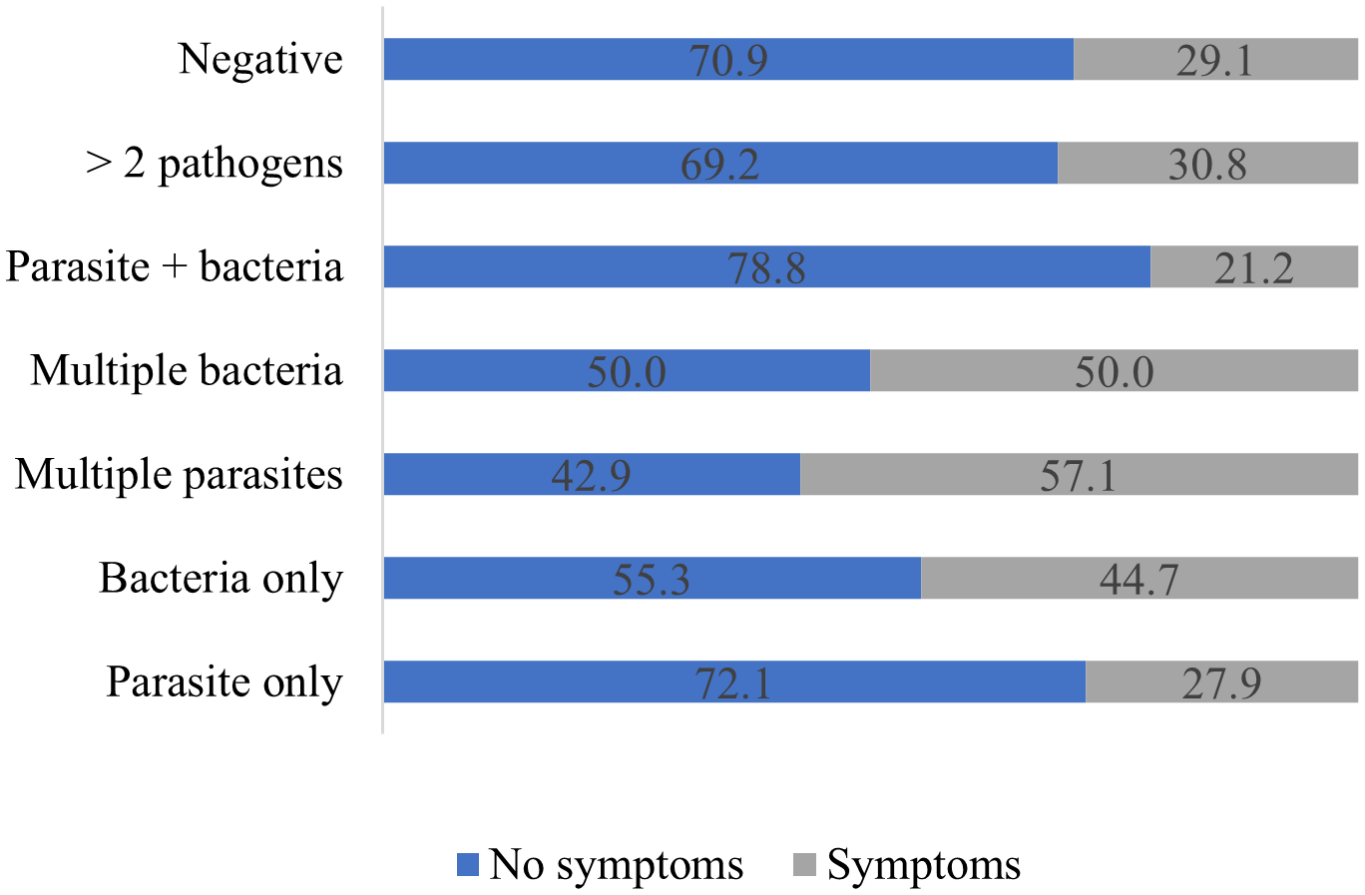
Intestinal infections among children by symptoms (n = 242), Madagascar 2025.

Clinical symptoms were reported in 106 children (43.8%), most commonly fever (n = 33), diarrhea (n = 30), abdominal pain (n = 23), rash or itching (n = 14), and constipation (n = 5). However, a large proportion of children remained asymptomatic despite laboratory-confirmed intestinal infections. Notably, asymptomatic carriage was observed even in children with mixed parasitic–bacterial infections. The highest proportion of symptomatic cases occurred among children infected with multiple parasite species (Fig 5).

Analysis of the association between infection type and intestinal symptoms showed a statistically significant correlation (Pearson’s chi-square test: 𝑋² = 341.68; df = 16, p < 2.2 × 10⁻¹⁶). The high chi-square value likely reflects substantial heterogeneity and sparse distribution across multiple infection–symptom categories rather than a single dominant association.

Hospitalization due to persistent diarrhea within the past three months was reported for two children (one with *Campylobacter* spp., one with no pathogen detected).

Fifteen children had recently received albendazole. Among these, *H. nana* was detected in 2 children and *G. intestinalis* in 6. Antibiotics had been administered to 16 children (11 amoxicillin, 3 metronidazole, 2 penicillin). One child treated with amoxicillin was positive for VTEC *E. coli*, and among those receiving metronidazole, one case of co-infection *with G. intestinalis* and *Blastocystis* spp. was identified.

Among children participating in regular deworming programs (n = 12; 5.0%), no helminth infections were detected, although *G. intestinalis* was present in 6 children. In the irregular deworming group (n = 43; 17.8%), *H. nana* (n = 3) and hookworm (n = 1) infections were identified, as well as *G. intestinalis* in 15 children, with sporadic cases of microsporidia and coccidia. In children who had not received deworming (n = 187; 59.7%), a total of 10 helminth infections were detected, including 4 hookworm, 5 *H. nana*, and 1 *T. trichiura*.

### Environmental and geographic factors

**Fig 6:**
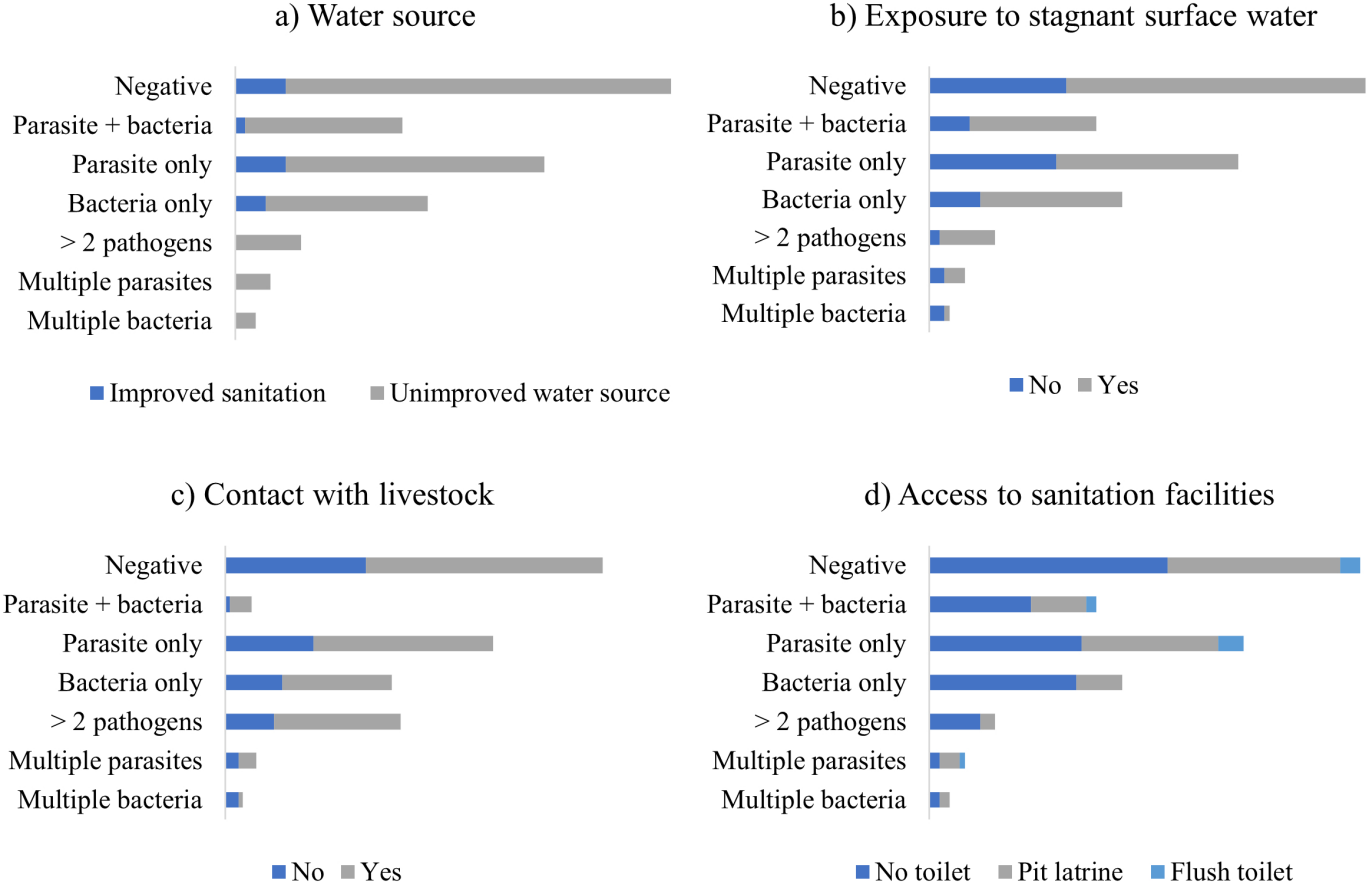
Association of environmental exposures with intestinal infection types: A: Water source; B: Exposure to stagnant Surface water; C: Contact with livestock; D: Access to sanitation facilities; Madagascar 2025.

The prevalence of intestinal infections varied according to environmental exposures and sanitation conditions. Multipathogen infections and co-infections were generally more frequent in children exposed to specific risk factors (Fig 6).

Only 28 children (11.6%) used piped water, while the majority (n = 214; 88.4%) relied on wells and/or surface water sources such as rivers and lakes. No multiple infections were observed among children with piped water access, except for two children with parasitic–bacterial co-infections. In this group, 10 children were infected only with parasites, 6 only with bacteria, and the remaining results were negative.

Exposure to stagnant surface water was reported for 164 children (67.8%) and was associated with a higher prevalence of infections other than single bacterial infections. Infections with more than two pathogens were notably more frequent among children exposed to stagnant water. In contrast, single bacterial infections were more common among children without such exposure.

Contact with livestock was reported by 159 children (65.7%) and was associated with higher rates of multiple infections, parasitic–bacterial co-infections, and both parasitic and bacterial mono-infections compared to children without animal contact.

Lack of access to toilets affected 140 children (57.9%), 90 children (37.2%) used latrines, and only 12 children (5.0%) had access to flush toilets. Children without access to sanitation or using rudimentary facilities had a higher prevalence of bacterial infections. Among those using flush toilets, one child had a bacterial infection and six had parasitic infections.

**Fig 7:**
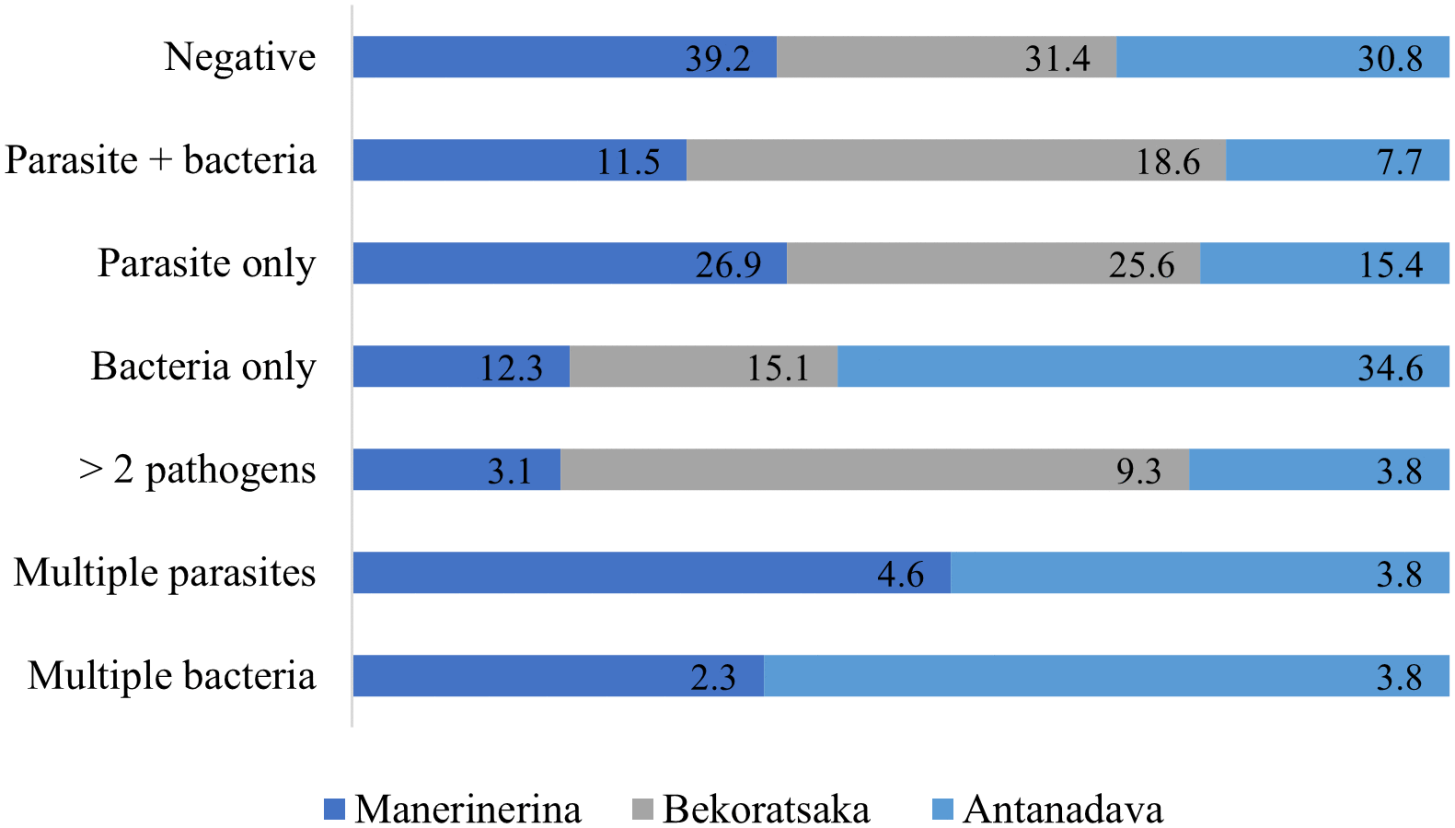
Intestinal infections among children by study sites (n = 242), Madagascar 2025.

Clear differences in the distribution of intestinal infections were observed between study sites (Fig 7). In Manerinerina, parasitic infections—both mono- and co-infections—dominated, with a high proportion of children free of detectable pathogens. Bekoratsaka had the highest proportion of complex infections involving multiple pathogens, including parasitic–bacterial co-infections. In Antanadava, bacterial infections were most common and represented the dominant infection category.

### Hematological profiles

Mean values of white blood cells (WBC), lymphocytes, granulocytes, hemoglobin (HGB), hematocrit (HCT), and platelets (PLT) were comparable across groups infected with different pathogens (Table 4). No statistically significant differences were observed between groups (Kruskal–Wallis test, all p > 0.05).

**Table 4:** Hematological parameters in children according to infection type (mean ± SD (95% CI)) (n = 242), Madagascar 2025.

	WBC	LYM	GRA	HGB	HCT	PLT
p-value *	0.1785	0.4015	0.5289	0.6286	0.5433	0.8678
**Multiple bacteria**	12 ± 7.4 (4.7-19.3)	6.4 ± 4.9 (1.6-11.2)	4.1 ± 1.9 (2.2-5.9)	11.8 ± 1.6 (10.3-13.3)	35.7 ± 3.2 (32.5-38.8)	352 ± 74 (279.5-424.5)
**Multiple parasites**	7.9 ± 3 (5.7-10.2)	4.4 ± 2.5 (2.5-6.2)	2.8 ± 0.8 (2.2-3.4)	11.2 ± 1 (10.5-12)	35.4 ± 2.8 (33.3-37.5)	386.3 ± 137.7 (284.2-488.3)
**>2 pathogens**	10.9 ± 4.1 (8.7-13.1)	5.5 ± 3.2 (3.7-7.2)	4.4 ± 2.6 (3-5.8)	10.9 ± 1.3 (10.2-11.7)	33.7 ± 3.2 (31.9-35.4)	372.6 ± 101.6 (317.4-427.8)
**Bacteria only**	8.9 ± 3.6 (7.7-10.1)	3.9 ± 1.9 (3.3-4.6)	4 ± 2.3 (3.3-4.8)	11.4 ± 1.3 (11-11.8)	35.6 ± 3.4 (34.5-36.7)	332.9 ± 103 (299.7-366.1)
**Parasite only**	8.1 ± 2.7 (7.5-8.8)	3.6 ± 1.4 (3.3-4)	3.6 ± 1.8 (3.2-4)	11.5 ± 1.5 (11.2-11.9)	35.6 ± 4.2 (34.5-36.6)	366 ± 104.2 (339.9-392.1)
**Parasite + bacteria**	9.4 ± 5.2 (7.6-11.2)	4.4 ± 3.4 (3.2-5.5)	4.1 ± 1.9 (3.4-4.7)	11.6 ± 1.5 (11.1-12.1)	36 ± 3.9 (34.7-37.3)	359.2 ± 105.8 (323.1-395.3)
**Negative**	8.4 ± 2.5 (7.9-9)	3.9 ± 1.6 (3.5-4.2	3.7 ± 1.6 (3.3-4)	11.4 ± 1.4 (11.1-11.7)	35.3 ± 3.8 (34.5-36.1)	345.9 ± 97.3 (325.4-366.5

* Kruskal-Wallis rank sum test

Risk factors for intestinal infectionsEpidemiological analysis indicated a high prevalence of intestinal infections in the study population. No significant differences in the proportion of children with any infection were observed according to water source, sanitation conditions, exposure to stagnant surface water, contact with livestock, sex, or age group (all Fisher’s exact tests, p > 0.05; odds ratios and 95% confidence intervals are presented in Table 5). Multivariable logistic regression for overall infection status did not identify any independent predictors included in the model, and the estimation of odds ratios for sanitation variables was limited by quasi-complete separation of categories.

**Table 5:** Fisher’s exact test results for any intestinal infection according to demographic and environmental factors in the study population.

Variable	OR	95% CI	p-value
**Water (well/river vs improved)**	1.12	0.45–2.67	0.837
**Sanitation (None/Pit vs Flush)**	–	–	0.819
**Contact with livestock (Y/N)**	1.22	0.68–2.19	0.483
**Stagnant surface water (Y/N)**	0.97	0.53–1.77	1.000
**Sex (M/F)**	1.33	0.76–2.35	0.347
**Age (<5 lat = ref)**	–	–	0.112

Notes: OR = odds ratio relative to the reference category. Missing OR and CI values for some categories reflect limitations due to quasi-complete separation. *No correction for multiple testing was applied; results are exploratory.*

When infection complexity (mono- vs. co-infection) was considered, environmental and demographic associations emerged. Children using unimproved water sources (well/river) had significantly higher odds of co-infections or multi-pathogen infections compared with children using improved sources (adjusted OR = 0.19, 95% CI: 0.03–0.75). Additionally, children aged 5–10 years exhibited a significantly higher odds of complex infections than younger children (<5 years, reference group; adjusted OR = 2.26, 95% CI: 1.07–4.86). No statistically significant associations were observed for sex, type of sanitation, livestock contact, or exposure to stagnant surface water in the multivariable model (Table 6). These results suggest that while environmental and demographic factors were associated with infection complexity, whereas no association was observed for overall infection presence, highlighting the importance of assessing co-infections in epidemiological studies. The distribution of mono- and co-infection cases across exposure categories is presented in S4 Table.

**Table 6:** Multivariable logistic regression for intestinal infection complexity (mono- vs. co-infection).

Variable	OR	95% CI	p-value
**Intercept**	2.53	0.31–28.35	0.405
**Age 5–10 vs <5 years**	2.26	1.07–4.86	0.035
**Age >10 vs <5 years**	0.61	0.28–1.30	0.327
**Water (well/river vs improved)**	0.19	0.03–0.75	0.037
**Sex (M vs F)**	1.08	0.52–2.22	0.835
**Sanitation: None vs Flush**	2.55	0.40–15.93	0.305
**Sanitation: Pit vs Flush**	2.29	0.36–14.12	0.359
**Contact with livestock (Y/N)**	1.04	0.45–2.37	0.932
**Stagnant surface water (Y/N)**	0.74	0.29–1.85	0.516

Notes: OR = adjusted odds ratio; CI = 95% confidence interval. Reference groups are indicated in the variable description. *No correction for multiple testing was applied; results are exploratory.*

## Discussion

In the studied population of children from northern Madagascar, a high prevalence of intestinal pathogens was observed, consistent with regions of limited sanitation infrastructure, where exposure to enteric microorganisms begins early and is nearly continuous. Data from Madagascar consistently indicate that parasitic infections—particularly protozoan infections—dominate the local epidemiological landscape and frequently coexist with bacterial enteropathogens. In the poorest districts of Antananarivo, nearly all children aged 2–5 years (96.3%) were infected with at least one intestinal parasite, with very high prevalence of *G. intestinalis* (79.5%), *Ascaris lumbricoides* (68.3%), and *T. trichiura* (68.0%) [26]. Over 90% of children exhibited polyparasitism, highlighting that single-pathogen infections are the exception rather than the rule. Similarly high prevalence (91.0%) has been reported in other regions, with a substantial proportion of co-infections involving at least two parasite species [18].

Against this background, findings from the Mampikony district appear to indicate lower infection rates. In 2023, clinically relevant intestinal parasites were detected in 27% of children aged 5–15 years, predominantly *G. intestinalis* (21.2%) [16], and in the following year, *G. intestinalis* and helminths were detected in 20% and 15% of children, respectively [17]. These differences may reflect not only true epidemiological variations but also methodological limitations, particularly underestimation of infections when relying solely on classical microscopy and the restricted scope of molecular diagnostics. The use of real-time PCR substantially increases pathogen detection, especially for low-intensity infections or the presence of biologically inactive parasite DNA [26], positioning molecular methods increasingly as a standard for epidemiological studies in populations with high asymptomatic carriage.

A notable aspect of the epidemiological picture was the high prevalence of potentially non-pathogenic parasites, such as *Blastocystis* spp. and non-pathogenic amoebae, also observed in other studies in Madagascar [16,17,27]. While these microorganisms rarely cause acute diarrhea, their presence serves as a reliable marker of fecal–oral transmission, typically via water, and indirectly reflects environmental hygiene [26]. Greigert et al. [28] found similar prevalence of most parasites among symptomatic and asymptomatic individuals, with only *G. intestinalis* significantly associated with diarrhea and abdominal discomfort.

Bacterial enteropathogens complement this epidemiological profile. Molecular studies among children with and without growth stunting in Antananarivo slums revealed extremely high carriage of *Shigella* spp., ETEC, EPEC, and EAEC, with these pathogens commonly present even in asymptomatic children [9]. Up to 93% of children carried at least one bacterial enteropathogen, and over one-third carried three or more simultaneously, despite the absence of acute diarrhea in the preceding period. These data underscore that lack of clinical symptoms does not imply low microbiological burden or absence of health consequences.

Co-occurrence of bacterial and protozoan infections is common in resource-limited settings. The presence of *G. intestinalis* alongside enteropathogenic bacteria such as *Campylobacter* spp. or pathogenic *E. coli* strains (STEC, EIEC) may modulate host immune responses and influence the clinical course of infection [13,14,27]. Some studies even suggest a protective effect of *Giardia* against acute diarrhea, related to induction of mucins and antimicrobial peptides that limit bacterial adherence to the intestinal epithelium. Conversely, highly invasive pathogens such as *Shigella*/EIEC or cytotoxic STEC may cause cumulative intestinal barrier damage, promoting environmental enteric dysfunction (EED), malnutrition, and growth impairment [9,29].

Collectively, these data indicate that children living in conditions of extreme resource limitation experience chronic, multifactorial exposure to enteric pathogens. The boundary between infection, carriage, and clinical disease is blurred, and the high prevalence of parasite–bacterial co-infections and asymptomatic infections highlights the limitations of classical symptom-based diagnostics. In this context, a positive PCR result in asymptomatic children should be interpreted as reflecting either subclinical infection or environmental exposure, indicating ongoing transmission within the community rather than serving as a standalone marker of clinical disease.The prevalence of intestinal parasite infections was strongly associated with environmental and sociodemographic factors, including access to safe water, sanitation, maternal education, and child age [26,30,31]. Consumption of untreated water remains a key risk factor for *G. intestinalis* infections, as observed in other regions of Africa and South America [31–33]. Children aged 5–10 years were at higher odds of infections due to increased mobility and frequent contact with contaminated environments [26,34]. No significant differences in infection prevalence were observed between boys and girls, consistent with other studies in Africa and Madagascar [26,35]. Age, however, emerged as a significant risk factor, with school-aged children more frequently infected with both intestinal parasites and bacterial enteropathogens. This pattern likely reflects both immunological changes and behavioral factors: greater autonomy, increased peer interactions, and more frequent exposure to contaminated water and soil [26,34,36,37].

Environmental factors are commonly implicated in shaping infection patterns in similar settings; however, no statistically significant associations were observed in the present study (Table 5). Nevertheless, lack of effective sanitation, frequent contact of children with livestock and their feces, and unstable water sources create conditions for continuous pathogen exposure. Environmental studies on Madagascar indicate that the main risk factor for *G. intestinalis* and other protozoa is the use of untreated or microbiologically contaminated water [26,32,33,38]. Water quality analyses revealed substantial variation in contamination with indicator bacteria depending on source type, location, and season, emphasizing that even urban water systems may exhibit significant microbiological fluctuations [39,40]. These findings are well established in the literature and our results are consistent with these previously described transmission pathways, confirming their persistence in a rural paediatric population in north-western Madagascar.

In this context, age and environment exposures were the main factors associated with intestinal infections in this study population.. School-aged children not only experience more frequent pathogen exposure but also have greater risk of sustained carriage and development of co-infections, amplified by widespread water contamination and inadequate sanitation infrastructure. Effective infection control strategies must therefore combine behavioral interventions with systemic improvements in WASH infrastructure.

Children in the studied regions remain at high risk due to poverty and unhygienic living conditions. Mass deworming campaigns and WASH interventions are essential to reduce the burden of parasitic and bacterial infections [25,41]. Standard albendazole-based programs remain the cornerstone for controlling intestinal helminths [16,28], but their efficacy against *H. nana* and protozoa, including *G. intestinalis*, is limited [26,33]. Persistent high prevalence of protozoan infections in regularly dewormed children highlights critical gaps in current preventive strategies.

Irrational antibiotic use presents an additional challenge. In the present study, one child with VTEC infection had received amoxicillin, highlighting that such practices also occur in the studied population.Field studies have reported treatment of VTEC-infected children with amoxicillin, which is not only ineffective but may promote resistance and disrupt gut microbiota [36]. Use of β-lactam antibiotics in VTEC infections is also associated with increased Shiga toxin release, potentially raising the risk of hemolytic–uremic syndrome [42]. Although clinical complications were not assessed in the present study, these observations underscore the importance of cautious antibiotic therapy where diagnostic capacity is limited.

The challenging realities of healthcare delivery in Madagascar must also be considered [15]. Microbiological diagnostics are available almost exclusively in major urban centers, and accessing a health facility often requires long, costly travel. In these circumstances, clinicians are forced to make therapeutic decisions under chronic diagnostic uncertainty. Empirical treatment, including antibiotics, often represents an attempt to safeguard the child, who may lack opportunity for follow-up. From this perspective, clinical decisions are not an expression of overprescription but a compromise imposed by systemic limitations.

These observations highlight the need for an integrated approach to control intestinal infections, combining pharmacotherapy with environmental interventions. Improved water quality, access to safe sanitation, and hygiene education are critical to reducing both single-pathogen infections and co-infections [26,40]. High rates of subclinical pathogen carriage demonstrate that absence of symptoms does not equate to absence of epidemiological risk or long-term health consequences, such as chronic intestinal inflammation or growth impairment [9,10].

Our findings confirm the high burden of intestinal infections and complex co-infections among children in rural Madagascar, dominated by *G. intestinalis* and *Campylobacter* spp. Environmental exposure, particularly use of unimproved water sources and contact with livestock, were associated with a higher prevalence of multi-pathogen infections. While these observations are consistent with previous studies conducted in Madagascar, including those by Collard et al. [9] and Habib et al. [26], our study provides additional evidence from a previously underrepresented rural population, characterized by high levels of co-infections and simultaneous exposure to multiple environmental risk factors. These results underscore the need to integrate routine deworming programs with safe water interventions, hygiene education, and strengthened local diagnostic and therapeutic capacities.

### Study limitations

This study has several important limitations that should be considered when interpreting the findings. First, no formal sample size calculation was performed, as the study was based on a convenience sampling approach driven by field feasibility. As a result, the study was not specifically powered for the analytical objectives, and some exposure categories in the regression analyses included relatively small numbers of participants, which may have affected the precision and stability of effect estimates. In addition, quasi-complete separation was observed for certain categorical variables, further limiting the stability of logistic regression estimates for specific exposures. Overall, the absence of a priori sample size calculation and the use of convenience sampling may limit the statistical power and generalisability of the observed associations.

Due to field conditions and limited resources, only a single stool sample per child could be collected. Three or more samples are recommended to increase sensitivity for detecting intestinal parasites, especially species shed intermittently [19]. Data collection was limited to a single month (August, dry season), which may not capture seasonal variation in intestinal pathogen transmission. Infection patterns in this setting may differ during the rainy season, when waterborne exposure is typically higher; therefore, the findings may not fully reflect year-round prevalence.

The heterogeneity of diagnostic methods is a limitation. Use of a commercial bacterial panel with inhibition control combined with in-house PCR assays for protozoa, lacking such control, may have influenced pathogen detection sensitivity. Moreover, PCR reactions were performed singly rather than in duplicates or triplicates, increasing the risk of random errors and false-negative results. In addition, these assays did not include an internal amplification or inhibition control, which may further affect the reliability of negative results, particularly in field-collected samples with variable matrix quality. Sample preservation (FTA or SAF/ethanol) and delayed analysis may also have affected DNA quality and pathogen detectability. Taken together, these methodological constraints may lead to an underestimation of true protozoan prevalence and should be considered when interpreting the reported prevalence estimates throughout the study. [43].

The inclusion of children with recent antibiotic exposure may have influenced bacterial detection rates and complicates interpretation of prevalence estimates. The small proportion of children using improved water sources limited the robustness of regression estimates for this variable and requires cautious interpretation. Furthermore, PCR-based detection does not allow differentiation between active infection and residual DNA, particularly in asymptomatic individuals, which may affect the interpretation of clinical relevance.

Limitations also stem from survey data: information on prior treatment, deworming, or sanitation practices may have been affected by recall bias or inaccurate caregiver reporting.The study population included children from selected localities, limiting representativeness and generalizability to other regions of Madagascar. Lack of full control over environmental factors, such as seasonal infection patterns, current water quality, or contact with wild animals, is another interpretive limitation. Furthermore, the small number of hospitalizations and prior treatments in the cohort limited assessment of treatment effects on intestinal infection prevalence.

Finally, the analysis of hematological parameters (Table 4) is limited by the wide age range of participants, small subgroup sizes, and lack of age-stratified reference values; therefore, it should be interpreted as exploratory rather than confirmatory.

Despite these limitations, the findings provide valuable data on infection prevalence, co-infections, and risk factors among children in rural Madagascar and can inform future research and public health interventions.

## Conclusions

Children from northern Madagascar are exposed to a high and chronic burden of intestinal pathogens, including both parasites and bacterial enteropathogens. High co-infection rates and frequent asymptomatic carriage observed in this study suggest that simultaneous exposure tomultiple pathogens is common in this setting, and the boundary between infection and clinical disease is not always clearly defined.. Pathogen exposure appears to be largely determined by environmental conditions—use of untreated water and lack of sanitation.

While these findings are consistent with previous studies, this work provides additional data from a previously underrepresented rural population, particularly regarding co-infection patterns and combined environmental exposures. The findings also highlight the presence of subclinical infections, which, despite lack of symptoms, may have epidemiological significance and and warrant further investigation in terms of their clinical impact.. These observations underscore the need for integrated public health strategies combining preventive measures, improved sanitation, and hygiene education rather than relying solely on symptomatic treatment or single deworming programs.

## Supporting information

S1 Data Dataset of study participants and variables included in the analysis. The dataset contains anonymized individual-level data for 242 children included in the study. Variables include participant ID, age group (<5, 5–10, >10 years), sex, place of residence (Manerinerina, Bekoratsaka, Antanadava), infection status (no infection, single infection, co-infection, multiple infections), presence of gastrointestinal symptoms, environmental exposures (water source, exposure to stagnant surface water, contact with livestock, sanitation access), and hematological parameters, including white blood cell count (WBC), lymphocytes (Limf), granulocytes (Gran), hemoglobin (HGB), hematocrit (HCT), and platelet count (PLT).
(CSV)

S1 Table MIQE assay parameter checklist for commercial quantitative PCR platforms used in this study. (DOCX)

S2 Table Frequency of intestinal parasite infections among children (n = 242). (DOCX)

S3 Table Frequency of gastrointestinal bacterial infections among children (n = 242) (monoinfections and coinfections), Madagascar 2025. (DOCX)

S4 Table Distribution of mono- and co-infections across exposure categories. (DOCX)
