# Correction: Chronic exposure to intestinal parasites and bacterial enteropathogens among children in rural Madagascar: Implications for asymptomatic carriage and co-infections

**DOI:** 10.1371/journal.pntd.0014718

**Published:** 2026-09-14

**Authors:** Wanesa Wilczyńska, Daniel Kasprowicz, Krzysztof Korzeniewski

The ORCID iDs are missing for the second and third author. Please see the authors’ respective ORCID iDs here:

- Author Daniel Kasprowicz’s ORCID iD is: 0000-0001-6357-2418 (https://orcid.org/0000-0001-6357-2418)
- Author Krzysztof Korzeniewski’s ORCID iD is: 0000-0002-0505-6279 (https://orcid.org/0000-0002-0505-6279)
