# Supplementary material for: Chronic exposure to intestinal parasites and bacterial enteropathogens among children in rural Madagascar: Implications for asymptomatic carriage and co-infections
Source: PLoS Negl Trop Dis. 2026 Jul 7;20(7):e0014519. doi: 10.1371/journal.pntd.0014519 (PMC13367895; doi:10.1371/journal.pntd.0014519)
Supplement: S1 Table — (DOCX) [file pntd.0014519.s002.docx]

**Table S1. MIQE assay parameter checklist forcommercial quantitative PCR platforms used in this study.**

| MIQE Parameter | AmpliTest *Entamoeba histolytica* (Amplicon, Poland) | Bosphore Bacterial GI Panel Kit v1 (Anatolia Geneworks, Turkey) |
| --- | --- | --- |
| Assay Format | Triplex real-time PCR assay (optimized for 20 µL reaction volume) | Multiplex real-time PCR panel utilizing two distinct reaction mixtures (PCR Master Mix 1 and 2) |
| Application | Species-specific confirmatory test (differentiation of *E. histolytica* from *E. dispar*) | Screening and characterization of bacterial enteropathogens from stool samples |
| Target Organisms & Genes | *Entamoeba histolytica* (species-specific target), *Entamoeba dispar* (species-specific target) | **PCR Master Mix 1:** *Clostridium difficile* (toxin A/B, *tcdA/tcdB* genes); *Campylobacter* spp. (*C. jejuni, C. upsaliensis, C. coli, C. lari*; 16S rRNA gene); *Salmonella* spp. (*ttrB* gene)  **PCR Master Mix 2:** *Escherichia coli* VTEC/STEC (*stx-1/stx-2* genes); *Escherichia coli* EIEC / *Shigella* spp. (*ipaH* gene); *Yersinia enterocolitica* (*ail* gene) |
| Oligonucleotide Sequences | **Proprietary** (Confidential to the manufacturer) | **Proprietary** (Confidential to the manufacturer) |
| Fluorophores & Quenchers | **FAM**: *Entamoeba histolytica* DNA **Texas Red®**: *Entamoeba dispar* DNA | **PCR Master Mix 1:** **FAM**: *Clostridium difficile* toxin A/B  **HEX**: *Campylobacter* spp.  **Cy5**: *Salmonella* spp.  **PCR Master Mix 2:** **FAM**: *Escherichia coli* (VTEC/STEC) **HEX**: *Escherichia coli* (EIEC) / *Shigella* spp.  **Cy5**: *Yersinia enterocolitica* |
| Internal Control (IC) | Integrated internal control system (synthetic DNA component) amplified exclusively in the HEX channel | Integrated synthetic DNA molecule to monitor extraction efficiency and PCR inhibition; detected via Texas RED filters |
| Analytical Sensitivity (LOD) | Verified qualitatively as a diagnostic confirmatory test (per manufacturer protocol) | 95% positive cut-off limit of detection (copies/reaction): *C. difficile*: 29 |
| Controls Included | Positive Control (PC, engineered DNA template) and Negative Control (NC, water). Interpretation: Positive Cq ≤ 40 | Two multiplex Positive Controls (PC1 & PC2; synthetic DNAs) and Negative Control (dH₂O). Acceptance criteria: PC ≤ 28, IC ≤ 32 |
| Regulatory & Validation Status | Diagnostic kit for *in vitro* professional use managed under ISO 13485 quality management system | CE-IVD marked, certified *in vitro* diagnostic kit system managed under ISO 13485 |

All parameters and performance specifications for the commercial assays presented in this table were compiled directly from the official manufacturers' Instructions for Use (IFU) documents provided by the respective suppliers: AmpliTest Entamoeba histolytica (Amplicon sp. z o.o., Wrocław, Poland; Document Version 2.0.PL, published 01.03.2022) and Bosphore Bacterial GI Panel Kit v1 (Anatolia Tanı ve Biyoteknoloji A.Ş. / Anatolia Geneworks, Istanbul, Turkey; Document Code: MB541v3f, published 08.11.2023). Under MIQE guidelines for proprietary commercial diagnostic kits, independent verification of analytical sensitivity (LOD) and quality control parameters as stated by the certified manufacturer (ISO 13485) is considered acceptable for reporting transparency when exact oligonucleotide sequences are legally protected.
