# Supplementary material for: Chronic exposure to intestinal parasites and bacterial enteropathogens among children in rural Madagascar: Implications for asymptomatic carriage and co-infections
Source: PLoS Negl Trop Dis. 2026 Jul 7;20(7):e0014519. doi: 10.1371/journal.pntd.0014519 (PMC13367895; doi:10.1371/journal.pntd.0014519)
Supplement: S2 Table — (DOCX) [file pntd.0014519.s003.docx]

|  | | **Number of infected children (*n*)** | **Percentage of infected children (%)** | |
| --- | --- | --- | --- | --- |
| **HELMINTHS** | **14** | | **5.8** |  |
| **Hookworm** | 5 | | 2.1 |  |
| ***Hymenolepis nana*** | 8 | | 3.3 |  |
| ***Trichuris trichiura*** | 1 | | 0.4 |  |
| monoinfections | 3 | | 1.2 |  |
| co-infections | 11 | | 4.5 |  |
| + *G. intestinalis / G. intestinalis* + nonpathogenic parasites | 8 | | 3.3 |  |
| + nonpathogenic parasites | 3 | | 1.2 |  |
| **PATHOGENIC PARASITES** | **116** | | **47.9** |  |
| ***Giardia intestinalis*** | 105 | | 43.4 |  |
| monoinfections | 50 | | 20.7 |  |
| co-infections | 55 | | 22.7 |  |
| + coccidia | 1 | | 0.4 |  |
| + microsporidia | 4 | | 1.7 |  |
| + helminths | 8 | | 3.3 |  |
| + nonpathogenic parasites | 42 | | 17.4 |  |
| **Coccidia** | 4 | | 1.7 |  |
| *Cycloisospora belli* | 2 | | 0.8 |  |
| *Cyclospora cayetanensis* | 1 | | 0.4 |  |
| *Cryptosporidium* spp. | 1 | | 0.4 |  |
| monoinfections | 0 | | 0.0 |  |
| co-infections | 3 | | 1.2 |  |
| *C. cayetanensis* + *C. belli* | 1 | | 0.4 |  |
| *C. belli* + *Blastocystis* spp. | 1 | | 0.4 |  |
| *Cryptosporidium spp.* + *G. Intestinalis* | 1 | | 0.4 |  |
| **Microsporidium** | 7 | | 2.9 |  |
| *Enterocytozoon* spp. | 6 | | 2.5 |  |
| *Encephalitozoon* spp. | 1 | | 0.4 |  |
| monoinfections | 3 | | 1.2 |  |
| co-infections | 4 | | 1.7 |  |
| + *G. intestinalis*/ *G. intestinalis* + nonpathogenic parasites | 4 | | 1.7 |  |
| **NONPATHOGENIC PARASITES** | **132** | | **54.5** |  |
| *Blastocystis* spp. | 74 | | 30.6 |  |
| *Dientamoeba fragilis* | 2 | | 0.8 |  |
| *Entamoeba coli* | 33 | | 13.6 |  |
| *Entamoeba dispar* | 8 | | 3.3 |  |
| *Endolimax nana* | 15 | | 6.2 |  |

**Table S2. Frequency of intestinal parasite infections among children (n=242) (monoinfections and coinfections), Madagascar 2025.**
