# Supplementary material for: Chronic exposure to intestinal parasites and bacterial enteropathogens among children in rural Madagascar: Implications for asymptomatic carriage and co-infections
Source: PLoS Negl Trop Dis. 2026 Jul 7;20(7):e0014519. doi: 10.1371/journal.pntd.0014519 (PMC13367895; doi:10.1371/journal.pntd.0014519)
Supplement: S3 Table — (DOCX) [file pntd.0014519.s004.docx]

**Table S3. Frequency of gastrointestinal bacterial infections among children (n=242) (monoinfections and coinfections), Madagascar 2025.**

|  | **Number of infected children (*n*)** | **Percentage of infected children (%)** |
| --- | --- | --- |
| ***Salmonella* spp.** | 0 | 0.0 |
| ***Yersinia enterocolitica*** | 0 | 0.0 |
| ***Clostridium difficile*** | 0 | 0.0 |
| ***Campylobacter* spp.** | **45** | **18.6** |
| monoinfections | 9 | 3.7 |
| co-infections | 36 | 14.9 |
| + *E .coli* (VTEC) | 1 | 0.4 |
| + *E .coli* (VTEC) + *G. intestinalis +/-* nonpathogenic parasites | 3 | 1.2 |
| + E .coli (VTEC) + nonpathogenic parasites | 1 | 0.4 |
| + *E. coli* (EIEC)/*Shigella* | 1 | 0.4 |
| + *E. coli* (EIEC)/*Shigella* + coccidia + nonpathogenic parasites | 1 | 0.4 |
| + E. coli (EIEC)/*Shigella* + *G. intestinalis/ +/-* nonpathogenic parasites | 2 | 0.8 |
| + *E. coli* (EIEC)/*Shigella* + nonpathogenic parasites | 1 | 0.4 |
| + *E. coli* (EIEC)/*Shigella* + *E. coli* (VTEC)/ STEC + *G. intestinalis* + nonpathogenic parasites | 1 | 0.4 |
| +*G. intestinalis* +/- nonpathogenic parasites | 18 | 7.4 |
| + *G. intestinalis* + microsporidium + nonpathogenic parasites | 1 | 0.4 |
| + coccidia | 1 | 0.4 |
| + helminths + nonpathogenic parasites | 1 | 0.4 |
| + nonpathogenic parasites | 4 | 1.7 |
| ***E. coli* (VTEC)/(STEC)** | **17** | **7.0** |
| monoinfections | 2 | 0.8 |
| co-infections | 15 | 6.2 |
| + *E. coli* (EIEC)/*Shigella* + *G. intestinalis* + microsporidium + nonpathogenic parasites | 1 | 0.4 |
| + *Campylobacter* spp. | 1 | 0.4 |
| + *Campylobacter* spp. + *G. intestinalis* +/- nonpathogenic parasites | 3 | 1.2 |
| + *Campylobacter* spp. + nonpathogenic parasites | 1 | 0.4 |
| + *E. coli* (EIEC)/*Shigella* + *Campylobacter* spp. + *G. intestinalis* + nonpathogenic parasites | 1 | 0.4 |
| + *G. intestinalis* +/- nonpathogenic parasites | 5 | 2.1 |
| + pathogenic parasites | 3 | 1.2 |
| ***E. coli* (EIEC)/ *Shigella* spp** | **36** | **14.9** |
| monoinfections | 10 | 4.1 |
| co-infections | 26 | 10.7 |
| + *Campylobacter spp.* | 1 | 0.4 |
| + Campylobacter spp. + coccidia + nonpathogenic parasites | 1 | 0.4 |
| + Campylobacter spp. + *G. intestinalis +/-* nonpathogenic parasites | 2 | 0.8 |
| + *Campylobacter spp.*  + nonpathogenic parasites | 1 | 0.4 |
| + *Campylobacter* spp. + *E. coli* (VTEC)/ STEC + *G. intestinalis* + nonpathogenic parasites | 1 | 0.4 |
| + *E .coli* (VTEC) + *G. intestinalis* + microsporidium + nonpathogenic parasites | 1 | 0.4 |
| *+ G. intestinalis* +/- nonpathogenic parasites | 8 | 3.3 |
| + helminths *+ G. intestinalis* +/- nonpathogenic parasites | 2 | 0.8 |
| + *G. intestinalis* + microsporidium | 1 | 0.4 |
| + nonpathogenic parasites | 8 | 3.3 |
