# Supplementary material for: Chronic exposure to intestinal parasites and bacterial enteropathogens among children in rural Madagascar: Implications for asymptomatic carriage and co-infections
Source: PLoS Negl Trop Dis. 2026 Jul 7;20(7):e0014519. doi: 10.1371/journal.pntd.0014519 (PMC13367895; doi:10.1371/journal.pntd.0014519)
Supplement: S4 Table — (DOCX) [file pntd.0014519.s005.docx]

| Variable | Category | Mono-infection | | Co-infection | | Total |
| --- | --- | --- | --- | --- | --- | --- |
|  |  | **n** | **(%)** | **n** | **(%)** | **n** |
| Water | Improved | 16 | 57.1 | 2 | 7.1 | 28 |
| Water | Well/river | 83 | 38.8 | 14 | 6.5 | 214 |
| Sex | Male | 54 | 44.6 | 28 | 23.1 | 121 |
| Sex | Female | 29 | 24.0 | 45 | 37.2 | 121 |
| Age | <5 | 32 | 34.4 | 27 | 29.0 | 93 |
| Age | 5–10 | 54 | 49.1 | 23 | 20.9 | 110 |
| Age | >10 | 13 | 33.3 | 7 | 17.9 | 39 |
| Sanitation | Flush | 4 | 36.4 | 3 | 27.3 | 11 |
| Sanitation | Pit | 36 | 40 | 20 | 22.2 | 90 |
| Sanitation | None | 59 | 41.8 | 34 | 24.1 | 141 |
| Contact with livestock | Yes | 66 | 41.5 | 39 | 24.5 | 159 |
| Contact with livestock | No | 33 | 39.8 | 18 | 21.7 | 83 |
| Stagnant surface water | Yes | 64 | 38.8 | 42 | 25.5 | 165 |
| Stagnant surface water | No | 35 | 45.5 | 15 | 19.5 | 77 |

**Table S4. Distribution of mono- and co-infections across exposure categories**
