# Supplementary material for: Chronic exposure to intestinal parasites and bacterial enteropathogens among children in rural Madagascar: Implications for asymptomatic carriage and co-infections
Source: PLoS Negl Trop Dis. 2026 Jul 7;20(7):e0014519. doi: 10.1371/journal.pntd.0014519 (PMC13367895; doi:10.1371/journal.pntd.0014519)
Supplement: Attachment — Submitted filename: RESPONSE TO REVIEWERS.docx [file pntd.0014519.s006.docx]

**RESPONSE TO REVIEWERS**

**Reviewer #1**

**Comment:** Methods are mostly clear. It is essential that the authors provide complete qMIQE details for all qPCR assays reported in the manuscript. qMIQE guidelines can be found here: https://pubmed.ncbi.nlm.nih.gov/19246619/. While details for the protozoan assays are mostly included, this information is missing for bacterial pathogens.

**Response:** We thank the Reviewer for this important comment. We have revised the Methods section. Specifically, we have added missing assay details for the bacterial panel, including target genes, amplification conditions, and information on controls and cycle threshold value. These additions have been incorporated into the revised manuscript to improve transparency and reproducibility.

**Comment:** The authors do not describe a clear justification or rationale for why the specific pathogens they measured were selected (and not others).

**Response:** We thank the Reviewer for this comment. We have added a clarifying statement in the Methods section explaining the rationale for the selection of bacterial pathogens included in this study. The selected panel targets the most clinically relevant and globally recognized bacterial causes of paediatric diarrhoeal disease, as supported by WHO guidance. This has now been explicitly stated and referenced in the revised manuscript (World Health Organization, Diarrhoeal disease fact sheet).

**Comment:** No attempt is made to provide quantitative estimates of targets, which is a major limitation of the paper. Given the use of qPCR, these results should be available, and the relative concentrations of targets could help with interpretation of their potential clinical relevance, as explained in this paper: https://www.thelancet.com/journals/lancet/article/PIIS0140-6736(16)31529-X/abstract.

**Response:** We thank the reviewer for this insightful suggestion. We agree that quantitative qPCR approaches, such as those applied in large-scale enteric disease studies (e.g. GEMS), can provide important additional resolution regarding pathogen burden and potential clinical relevance. However, we would like to clarify that the primary aim of our study was not quantitative pathogen attribution, but rather cross-sectional detection of enteric pathogens in a rural paediatric population including both symptomatic and asymptomatic children. In this context, the study was designed to assess presence/absence patterns rather than to model pathogen load or disease causality.

Importantly, the study was conducted using dried stool spots (filter paper–based specimens), which imposes additional methodological constraints for robust quantitative qPCR interpretation, particularly in the absence of validated conversion frameworks for absolute or relative quantification in this matrix. We fully agree that adaptation of quantitative qPCR approaches to such field-compatible sample types would be highly valuable. This represents an interesting and promising direction for future work, particularly following dedicated methodological validation studies for filter paper–based diagnostics.

**Comment:** This paper is a well-written, basic analysis of enteric pathogens in children in an understudied context. Although its central insights are not new - high prevalence of enteric pathogen detection in stool from asymptomatic children in high-burden settings is the norm and this has been known for years - it is a useful study that may be of general interest.

**Response:** We thank the reviewer for the positive and constructive assessment of our manuscript and for recognizing its relevance in an understudied setting. We greatly appreciate the overall supportive evaluation and helpful feedback provided.

**Reviewer #2**

**Comment:** The study objectives are partially articulated. The aim stated at lines 99–103 identifies prevalence estimation, asymptomatic carriage, co-infections, and environmental associations as targets, but no formal testable hypothesis is stated. The inclusion of haematological parameters in the objectives appears as an afterthought with no hypothesis attached.

**Response:** We thank the Reviewer for this valuable suggestion. We have revised the objectives to include explicit study hypotheses. In particular, we now state that we hypothesised an association between intestinal pathogen carriage and environmental exposure factors, and that infected children may exhibit alterations in selected haematological parameters reflecting systemic inflammatory response.

**Comment:** A community-based cross-sectional design is appropriate for estimating prevalence and describing co-infection patterns in a defined paediatric population. However, the design is not adequate to support causal or directional claims about risk factors, which the authors make in both the results and discussion.

**Response:** We thank the reviewer for this important comment. We agree that, given the cross-sectional design of the study, causal or directional interpretations are not appropriate. We have therefore revised the manuscript throughout the Results and Discussion sections to replace causal language with appropriate terms. We have also carefully rephrased interpretations of environmental and demographic variables to avoid implying causality beyond the study design.

Below are the sentences that have been revised accordingly:

- These results suggest that while environmental and demographic factors do not significantly influence overall infection presence, they may contribute to multi-pathogen infections 🡪 These results suggest that while environmental and demographic factors **were associated with infection complexity, whereas no association was observed for overall infection presence**

- higher **risk** of complex infections 🡪 higher **odds** of complex infections

- age and environment act synergistically as the main determinants of intestinal infections 🡪 age and environmental exposures were the main factors **associated** with intestinal infections in this study population.

- Environmental determinants largely drive these predispositions. 🡪 Environmental factors are commonly implicated in shaping infection patterns in similar settings…

- Environmental exposure, particularly use of unimproved water sources and contact with livestock,promote multi-pathogen infections 🡪 Environmental exposure, particularly use of unimproved water sources and contact with livestock, **were associated** with a higher prevalence of multi-pathogen infections

**Comment:** The study population is inadequately described at the point where results are presented. No summary table of participant characteristics is provided before the infection data, making it impossible for the reader to interpret the findings in context. Age distribution is mentioned in passing in the results narrative rather than presented systematically. Sex, site distribution, water source, sanitation access, livestock contact, and antibiotic or deworming exposure are scattered across the text rather than consolidated.

**Response:** We thank the reviewer for this valuable suggestion. In response, we have added a new Table 2 summarising the socio-demographic and environmental characteristics of the study population. This table is now presented at the beginning of the Results section to provide a clear overview of the study cohort and improve readability and context for subsequent analyses.

**Comment:** No sample size calculation is provided. The study enrolled 242 children, described implicitly as a convenience sample, with no justification of statistical power for any of the stated objectives. This is a significant gap. For the logistic regression analyses in Tables 5 and 6, several exposure subgroups are critically small, particularly the improved water source group (n=28), rendering regression estimates unstable and the associated confidence intervals unreliable. The authors must either provide a post hoc power calculation or explicitly acknowledge that the study is underpowered for analytical objectives and limit conclusions accordingly.

**Response:** We acknowledge that no formal sample size calculation was performed, as the study was designed as an observational cross-sectional survey based on a convenience sample of children attending participating sites during the study period. The sample size was therefore determined by feasibility and field conditions rather than predefined statistical power considerations.

We agree that several exposure categories in the regression analyses, particularly the improved water source group, include relatively small numbers of participants, which may affect the precision and stability of the estimated associations.

We have therefore revised the manuscript to explicitly acknowledge this limitation in the Study Limitations section and have moderated the interpretation of regression results in both the Results and Discussion sections to avoid overstatement of inferential conclusions.

**Comment:** Fisher's exact test and chi-square tests are applied appropriately for categorical comparisons, and the Kruskal-Wallis test is correctly used for non-parametric haematological data. However, no correction for multiple comparisons is applied despite the large number of statistical tests performed across infection categories, environmental variables, and haematological parameters, raising the risk of type I error throughout. The logistic regression for infection complexity is limited by quasi-complete separation for sanitation variables, which the authors note but do not adequately address. No false discovery rate adjustment is reported. The heading "Statistical Analysis of Infection Prevalence and Risk Factors" appears within the results section at line 401, which is a structural error. The Pearson chi-square value reported at line 343 (X² = 341.68, df = 16) is extremely large relative to the sample size of 242.

**Response:** We thank the reviewer for this careful statistical assessment. We have revised the manuscript to address these concerns. The Results section heading has been corrected to a descriptive format, and we have clarified in the Methods that no adjustment for multiple comparisons (including FDR) was applied due to the exploratory nature of the analyses, which is now also reflected in the Limitations. We further acknowledge limitations related to sparse data and quasi-complete separation affecting some logistic regression estimates, particularly for sanitation variables. Finally, we added an interpretative note explaining that the high chi-square value reflects heterogeneity and sparse distribution across categories rather than a single dominant association.

**Comment:** Lines 100-103: rationale to include haematological parameters not stated. Why were CBC parameters hypothesized to differ by infection type in an asymptomatic community cohort? Provide justification with a biological rationale and supported by prior literature before data collection.

**Response:** We thank the reviewer for this important comment. We have now clarified the rationale for including haematological parameters in the Introduction section. Although the study population was largely asymptomatic, chronic and repeated exposure to intestinal pathogens in such settings is known to induce subclinical inflammation, immune activation, and nutritional consequences, including anemia. Previous studies have shown that both parasitic and bacterial infections may be associated with alterations in hematological parameters such as leukocyte counts, differential cell profiles, and hemoglobin levels, even in the absence of overt clinical symptoms. We have added appropriate references and clarified that the analysis of CBC parameters was exploratory and aimed at assessing potential subclinical systemic effects of infection.

**Comment:** Line 107: states all samples were collected in August 2025. Were all 242 samples truly collected within a single month? If so, this must be explicitly stated and its implications for representativeness discussed, particularly given seasonal variation in infection patterns.

**Response:** We confirm that all samples and questionnaire data were collected within a single field campaign conducted in August 2025. This has now been explicitly stated in the Methods section. We acknowledge that this cross-sectional design, limited to the dry season, may not capture seasonal variation in intestinal pathogen transmission. To address this, we have added a statement in the Limitations section noting that infection patterns may differ during the rainy season and that the findings may not fully reflect year-round prevalence.

**Comment:** Lines 125-126: both symptomatic and asymptomatic children were included regardless of recent antimicrobial use. were the included samples diarrheal or non-diarrheal? The bacterial panel used is validated for diarrheal stool. The implications of applying it to non-diarrheal samples must be addressed.

**Response:** We appreciate this important comment. The study population included both diarrhoeal and non-diarrhoeal stool samples, as the study was designed to assess not only clinically manifest infections but also asymptomatic carriage and exposure to enteric pathogens in a high-transmission setting. While the applied molecular panel is primarily validated for diarrhoeal samples, it targets pathogens of well-established epidemiological relevance in paediatric populations. Importantly, in low-resource and high-endemicity settings, several enteropathogens traditionally associated with diarrhoea, including *Giardia intestinalis* and *Cryptosporidium* spp., are frequently detected in asymptomatic individuals, reflecting ongoing exposure and transmission rather than overt disease.

Therefore, the use of this panel in non-diarrhoeal samples was considered appropriate for the broader epidemiological objective of the study. To address this point, we have now clarified the rationale for panel selection in the Method section.

**Comment:** Line 220: Ct cutoff of 38 is used- reference justifying this threshold for each pathogen and this sample matrix (filter paper eluate) is required.

**Response:** We thank the reviewer for this important methodological point. The Ct cut-off of 38 was selected in the context of a 45-cycle amplification protocol and was based on empirical analysis of amplification curves obtained in this study. Signals appearing after cycle 38 consistently demonstrated non-exponential and unstable fluorescence patterns, suggesting reduced reliability of amplification at later cycles.

In addition, the use of filter paper–derived stool eluates represents a low-yield and complex matrix, where late amplification signals are more likely to reflect non-specific amplification or minimal template artefacts. In the absence of matrix-specific external quantitative standards, a conservative Ct threshold was therefore applied to reduce the risk of false-positive interpretation. This justification has now been clarified in the Methods section.The Ct cutoff of 32 used for interpretation of the bacterial multiplex panel was not arbitrarily selected. It was defined in accordance with the manufacturer’s instructions for use (IFU), which specify that internal control (IC) amplification above Ct 32 indicates suboptimal amplification or potential inhibition, and therefore invalid or unreliable assay performance. In our study conditions (40 amplification cycles), the internal control consistently amplified within the acceptable range, and the positive control reproducibly yielded Ct values around 28, confirming correct assay performance and expected amplification efficiency. Accordingly, the Ct ≤ 32 threshold was applied as a conservative decision rule to ensure that only results obtained within the validated dynamic range of the assay were considered positive, while minimizing the risk of including late-cycle nonspecific amplification signals.

**Comment:** No sample size calculation is presented in methods. how 242 was determined, or explicitly acknowledge convenience sampling as a limitation with its impact on power.

**Response:** A formal sample size calculation was not performed a priori. The study was designed as a cross-sectional field investigation conducted under routine surveillance and logistical constraints, where the final sample size (n = 242) was determined by the number of eligible children available during the study period and consenting to participate.

We acknowledge that this approach corresponds to convenience sampling, which may limit statistical power for detecting small effect sizes and restrict the generalisability of the findings. This information has now been explicitly added to the Limitations section.

**Comment:** Lines 220-223: Only a single PCR reaction was performed per sample for the in-house protozoan assays, with no internal inhibition control. This is a methodological limitation that fundamentally affects the reliability of negative results. The authors acknowledge this briefly in limitations but do not adequately discuss how it affects interpretation of prevalence estimates throughout the paper.

**Response:** We agree that the use of single-run PCR reactions without an internal inhibition control represents a methodological limitation that may affect the reliability of negative results.

This limitation is particularly relevant in field-collected samples, where variable matrix quality and potential PCR inhibitors may lead to false-negative results, especially in low-intensity infections. We also acknowledge that this may result in an underestimation of the true prevalence of protozoan infections and therefore influence interpretation of the overall epidemiological estimates.

This point has now been strengthened and explicitly expanded in the Limitations section of the manuscript

**Comment:** The results section requires restructuring. It should begin with a table summarizing participant characteristics (Table 1 equivalent), including number of children under 5 and above, sex distribution, site, water source type, toilet access, livestock contact, and sanitation access stratified by relevant groupings. Without this, the infection data that follows lacks interpretive context. The reader cannot assess the representativeness or risk profile of the study population.

**Response:** We thank the reviewer for this helpful suggestion. A participant characteristics table (Table 2) has been added at the beginning of the Results section, including all recommended variables (age stratification, sex distribution, study site, water source type, sanitation access, toilet access, and livestock contact). We agree that this improves the interpretability of the infection-related analyses and the overall context of the study population.

**Comment:** "Improved sanitation" used in Fig 6a legend and throughout requires an operational definition.

**Response:** We thank the reviewer for this comment. The definition of “improved sanitation” has now been added to the Methods section under epidemiological and socio-economic data collection. It is aligned with WHO/UNICEF Joint Monitoring Programme criteria and now explicitly distinguishes improved and unimproved sanitation categories used in the analyses and figures.

**Comment:** Table 1 (primers): Rename appropriately so that the participant characteristics table becomes Table 1. Restructure so that primers are moved to supplementary material or renumbered accordingly.

**Response:** The primer table has been retained in the Methods section due to its methodological role and the presence of source references within the table content. However, we have ensured that participant characteristics are presented as the first table in the Results section to improve readability and narrative flow

**Comment:** Tables 2 and 3 (frequency of infections): Given the small sample size, the level of sub-stratification in these tables is not statistically supportable. Many cells contain n=1 or n=2. These tables should be moved to supplementary material. A condensed summary table in the main text is sufficient.

**Response:** We thank the reviewer for this important suggestion. We agree that the level of stratification in the original Tables 2 and 3 resulted in sparse cell counts, limiting interpretability. The tables have therefore been moved to the Supplementary Material (Tables S1–S2).

In the revised manuscript, we have included a condensed summary table in the main text focusing on overall infection frequencies and key stratifications, in order to improve clarity and statistical interpretability.

**Comment:** Line 346: "Higher than expected" requires clarification. Expected under what model or assumption? State explicitly whether this refers to the independence model or a clinical expectation and provide the statistical basis.

**Response:** We thank the reviewer for this comment. The statement referring to expected values under the independence model and Pearson residuals has been removed to improve clarity and avoid overinterpretation, as it was not essential for the main conclusions of the study.

**Comment:** Line 349: Children who recently received antibiotics were included in the analysis of bacterial infections. If antibiotic exposure within 28 days preceded sample collection, bacterial detection rates are not interpretable. Were these children excluded from bacterial prevalence analysis?

**Response:** We thank the reviewer for this important comment. Children who had received antibiotics within the preceding 28 days were not excluded from the bacterial analysis, as antibiotic exposure was not used as an exclusion criterion in the study design. Given the small number of antibiotic-exposed participants (n=16), no stratified or adjusted analysis was performed. The relevant results are reported descriptively in the Results section.

**Comment:** Also define "multipathogen infection" at first use with a clear operational definition (e.g., detection of two or more distinct pathogen species by any method).

**Response:** We thank the reviewer for this comment. We have now added a clear operational definition of multi-pathogen infection at first mention in the Results section. Multi-pathogen infection is defined as the detection of two or more distinct pathogen species within a single specimen, regardless of taxonomic group. The terminology has also been corrected throughout the manuscript.

**Comment:** Table 4: This table is scientifically weak as presented. No sig differences, and infection subgroups include cells with very small numbers (e.g., multiple bacteria n=4). The age range of participants spans infants to adolescents, a group in which CBC reference ranges differ substantially. WBC, lymphocyte counts, and platelet values are not comparable across such a wide age range without age-stratified analysis or adjustment. There is no a priori biological justification for expecting CBC parameters to differ by infection type in an asymptomatic cohort. The authors must either justify the analysis with a clear hypothesis and age-adjusted approach.

**Response:** We thank the reviewer for this insightful comment. We agree that the interpretation of hematological parameters is limited by the wide age range of the study population and the absence of age-stratified analysis. This analysis was intended as an exploratory, hypothesis-generating assessment rather than a confirmatory evaluation of biologically expected differences between infection groups.

We have clarified this in the manuscript and explicitly acknowledge that the small subgroup sizes and lack of age adjustment limit the interpretability of these findings. We also agree that no strong a priori biological hypothesis was assumed for differences in CBC parameters across infection categories in this asymptomatic cohort.

**Comment:** No anthropometric measurements (height, weight, MUAC, or stunting status) are presented. In a paediatric cohort in a food-insecure region where environmental enteric dysfunction is a stated concern, this is a significant omission. Were these data collected? If so, they should be included and analyzed in relation to infection status.

**Response:** Anthropometric measurements (including height, weight, MUAC, and stunting assessment) were not collected during this field study due to logistical and resource constraints. We acknowledge this as a limitation, as such data would have allowed a more comprehensive assessment of the relationship between nutritional status, environmental enteric dysfunction, and infection patterns in this cohort.

**Comment:** Table 5: The take-home message is unclear. The table shows no significant associations for any variable with overall infection status, yet the discussion draws conclusions about environmental risk factors. The authors must reconcile this null result with their interpretive claims and explain what Table 5 contributes to the paper.

**Response:** We thank the reviewer for this important comment. We agree that Table 5 shows no statistically significant associations between the evaluated environmental and demographic factors and overall infection status. We have revised the Discussion to avoid overstating these findings and to clarify that these analyses were exploratory and did not demonstrate significant risk factor associations in this dataset. The role of Table 5 has been clarified as providing descriptive context rather than confirming independent predictors of infection.

**Comment:** Table 6: The inference drawn from this table is limited by the very small number of children using improved water sources (n=28, 11.6%). An adjusted OR of 0.19 from a subgroup of 28 individuals should be interpreted with extreme caution. This limitation must be prominently acknowledged. Additionally, the number of children in mono-infection versus co-infection groups for each exposure variable must be stated in the table or footnote to allow the reader to assess the reliability of the estimates.

**Response:** No statistically significant associations were observed for sex, type of sanitation, livestock contact, or exposure to stagnant surface water in the multivariable model (Table 66). These results suggest that while environmental and demographic factors were associated with infection complexity, whereas no association was observed for overall infection presence do not significantly influence overall infection presence, they may contribute to multi-pathogen infections, highlighting the importance of assessing co-infections in epidemiological studies. The distribution of mono- and co-infection cases across exposure categories is presented in Supplementary Table S3.

**Comment:** Lines 442 onwards: The discussion of environmental risk factors does not address weather or seasonality, despite the fact that all sampling occurred during the dry season. Infection patterns for enteric pathogens, particularly Giardia, Campylobacter, and EIEC, are known to vary by season. The absence of wet season data is a significant limitation that must be discussed here explicitly.

**Response:** We have addressed this point by explicitly acknowledging in the Limitation Section that all samples were collected during the dry season, which may influence enteric pathogen transmission dynamics and limits comparison with other seasonal periods..

**Comment:** Line 480: The statement that a positive PCR result should be interpreted as an indicator of environmental exposure rather than disease is scientifically problematic as written. PCR positivity in an asymptomatic child does not negate clinical relevance. The authors must clarify: are they suggesting these children should not be treated? If PCR is positioned purely as an environmental indicator, the clinical management implications for individual children must be addressed directly. The framing as written seems misleading.

**Response:** We agree that PCR positivity should not be interpreted as lacking clinical relevance. We have revised the statement to clarify that PCR detection in asymptomatic children may reflect either subclinical infection or environmental exposure, indicating ongoing transmission rather than serving as a standalone marker of clinical disease. We have avoided any implication regarding treatment decisions.

**Comment:** Line 495: The findings described as novel here (environmental risk factors driving infection, unimproved water associated with parasitic infections, livestock contact associated with bacterial carriage) are well-established including from Madagascar specifically. The authors must demonstrate explicitly how their findings extend or challenge current knowledge rather than restate what is already known.

**Response:** We acknowledge that the environmental determinants discussed are well established in the literature, including in Madagascar. We have revised the text to avoid implying novelty and to clarify that our findings confirm previously described transmission pathways in this setting. Importantly, our study contributes additional empirical data from a previously under-studied rural paediatric population in north-western Madagascar, thereby strengthening the evidence base for the persistence of these transmission routes in similar resource-limited settings.

**Comment:** Line 504: The discussion references school-aged children and behavioral exposure patterns related to school attendance. Were study participants attending school? If so, school attendance and hygiene practices at school should be addressed as a potential exposure and a potential intervention point. If this information was not collected, that is a limitation.

**Response:** We agree that school attendance and hygiene practices may represent relevant exposure pathways. However, these data were not collected in the present study and therefore could not be analysed. This has been acknowledged as a limitation, and future studies should consider including school-related exposures as potential determinants of infection risk.

**Comment:** Line 516: The discussion of irrational antibiotic use and VTEC management references a field study finding but does not connect this to the authors' own data. The authors found one child treated with amoxicillin who was positive for VTEC. This is the relevant result to discuss here. The paragraph must be grounded in the authors' findings, not external observations alone.

**Response:** We thank the reviewer for this important comment. We agree that the discussion should be more explicitly grounded in the study findings. We have therefore revised this paragraph to directly incorporate our own observation of one child with VTEC infection who had received amoxicillin, and we used this finding as an entry point to contextualize antibiotic use practices with respect to published evidence on VTEC management.

**Comment:** Line 530: The claim that high subclinical carriage rates demonstrate epidemiological risk independent of symptoms is not new and is not uniquely supported by this dataset. The authors must articulate what this study adds to what is already established by Collard et al. 2022 and Habib et al. 2021, both of which are cited and report very similar findings from Madagascar.

**Response:** We agree that the role of subclinical carriage and environmental risk factors in intestinal infections in Madagascar has been previously described. We have revised the discussion to avoid overstating novelty and to explicitly position our findings as confirmatory evidence from a previously underrepresented rural population, with particular emphasis on the high burden of co-infections and combined exposure to multiple environmental risk factors.

**Comment:** No false discovery rate correction or other adjustment for multiple comparisons is reported despite numerous statistical tests being performed across infection categories, environmental variables, and haematological parameters. This must be addressed in the methods and results.

**Response:** We thank the reviewer for this important comment. We confirm that no correction for multiple testing was applied due to the exploratory nature of the analyses. This has been clarified in the Methods section and additionally stated in all relevant tables to ensure transparency in interpretation of the results.

**Comment:** The limitations section is incomplete. The following must be added:

• wide age range and its effect on CBC interpretation.

• absence of a wet season comparison.

• very small improved-water subgroup limiting the validity of regression estimates.

• inclusion of antibiotic-exposed children in bacterial prevalence analysis.

• inability to distinguish active infection from residual DNA in asymptomatic PCR-positive individuals.

Given the methodological limitations acknowledged by the authors themselves (single stool sample, single PCR reaction, no internal inhibition control for protozoan assays, convenience sample with no formal sample size calculation, extremely small subgroups for regression), the strength of the conclusions requires moderation throughout. The take-home message must be clearly stated: what does this study definitively add to the literature on intestinal co-infections in rural Madagascar that was not previously known?

**Response:** We thank the reviewer for this detailed and constructive comment. We have revised the limitations section to explicitly address the wide age range in relation to hematological parameters, the lack of wet season data, the small subgroup using improved water sources, the inclusion of children with recent antibiotic exposure, and the inability of PCR-based methods to distinguish active infection from residual DNA.

In addition, we have moderated the conclusions throughout the manuscript to better reflect the exploratory nature of the study and its methodological constraints. We have also clarified the specific contribution of this work as providing data from a previously underrepresented rural population, particularly with regard to co-infection patterns and combined environmental exposures.
