# Supplementary material for: Chronic exposure to intestinal parasites and bacterial enteropathogens among children in rural Madagascar: Implications for asymptomatic carriage and co-infections
Source: PLoS Negl Trop Dis. 2026 Jul 7;20(7):e0014519. doi: 10.1371/journal.pntd.0014519 (PMC13367895; doi:10.1371/journal.pntd.0014519)
Supplement: Attachment — Submitted filename: Response to reviewers (2).docx [file pntd.0014519.s007.docx]

Reviewer #1: Thank you for the response and for the revisions to the manuscript; it is much improved. However, apart from the protozoan targets, I do not see the full MIQE details for the assays used. Please refer to the MIQE guidelines: https://academic.oup.com/clinchem/article-abstract/55/4/611/5631762. A table in Supporting Information or an annex would be appropriate. While specifying the target genes is helpful, the actual sequences would also be useful for interpretation. If the specific sequences are unavailable because they are proprietary, that would be useful to note. If there are other studies that have validated the pathogen panel, they could be referenced as well.

**Response:** We sincerely thank the Reviewer for this highly constructive feedback and for the positive evaluation of our previous revisions. We fully agree that providing complete technical specifications for all molecular tools—including commercial ones—is essential for reproducibility, data interpretation, and compliance with the spirit of the MIQE guidelines.Following the Reviewer's excellent suggestions, we have implemented the following updates:

- Created a Dedicated MIQE Table (Supporting Information): We have compiled all available technical metadata for the commercial diagnostic systems into a new table, Table S1, now available in the Supporting Information. This table systematically itemizes the assay formats, multi-channel fluorophore/quencher allocations, internal control configurations, and analytical sensitivities (LOD).
- Addressed Proprietary Sequences: As correctly anticipated by the Reviewer, the exact primer and probe sequences for both AmpliTest and Bosphore panels are legally protected commercial properties. We have explicitly stated this limitation inside Table S1.
- Documented Source & Validation Background: As requested, we have added a comprehensive footnote under Table S1 clearly indicating that all data were compiled directly from the official manufacturers' technical documents (including specific document version codes and dates).
