# Supplementary material for: Chronic exposure to intestinal parasites and bacterial enteropathogens among children in rural Madagascar: Implications for asymptomatic carriage and co-infections
Source: PLoS Negl Trop Dis. 2026 Jul 7;20(7):e0014519. doi: 10.1371/journal.pntd.0014519 (PMC13367895; doi:10.1371/journal.pntd.0014519)
Supplement: Peer review history

# Peer review: Decision Letter 0

**DOI:** 10.1371/journal.pntd.0014519.r001

**Authors:** Joseph M. Vinetz, Luther A Bartelt

30 Apr 2026

PNTD-D-26-00325

Chronic exposure to intestinal parasites and bacterial enteropathogens among children in rural Madagascar: implications for asymptomatic carriage and co-infections

PLOS Neglected Tropical Diseases

Dear Dr. Wilczyńska,

Thank you for submitting your manuscript to PLOS Neglected Tropical Diseases. After careful consideration, we feel that it has merit but does not fully meet PLOS Neglected Tropical Diseases's publication criteria as it currently stands. Therefore, we invite you to submit a revised version of the manuscript that addresses the points raised during the review process.

Please submit your revised manuscript within by Jun 29 2026 11:59PM. If you will need more time than this to complete your revisions, please reply to this message or contact the journal office at plosntds@plos.org. When you're ready to submit your revision, log on to https://www.editorialmanager.com/pntd/ and select the 'Submissions Needing Revision' folder to locate your manuscript file.

Please include the following items when submitting your revised manuscript:

* A letter that responds to each point raised by the editor and reviewer(s). You should upload this letter as a separate file labeled 'Response to Reviewers'. This file does not need to include responses to any formatting updates and technical items listed in the 'Journal Requirements' section below.

* A marked-up copy of your manuscript that highlights changes made to the original version. You should upload this as a separate file labeled 'Revised Manuscript with Track Changes'.

* An unmarked version of your revised paper without tracked changes. You should upload this as a separate file labeled 'Manuscript'.

If you would like to make changes to your financial disclosure, competing interests statement, or data availability statement, please make these updates within the submission form at the time of resubmission. Guidelines for resubmitting your figure files are available below the reviewer comments at the end of this letter.

We look forward to receiving your revised manuscript.

Kind regards,

Luther A Bartelt

Academic Editor

PLOS Neglected Tropical Diseases

Joseph Vinetz

Section Editor

PLOS Neglected Tropical Diseases

Shaden Kamhawi

co-Editor-in-Chief

PLOS Neglected Tropical Diseases

orcid.org/0000-0003-4304-636XX

Paul Brindley

co-Editor-in-Chief

PLOS Neglected Tropical Diseases

orcid.org/0000-0003-1765-0002

**Journal Requirements:**

-->1) We have noticed that you have uploaded Supporting Information files, but you have not included a list of legends. Please add a full list of legends for your Supporting Information files after the references list.

2) Please ensure that the funders and grant numbers match between the Financial Disclosure field and the Funding Information tab in your submission form. Note that the funders must be provided in the same order in both places as well.

3) Please revise your current Competing Interest statement to the standard "The authors have declared that no competing interests exist."

4) Some material included in your submission may be copyrighted. According to PLOS’s copyright policy, authors who use figures or other material (e.g., graphics, clipart, maps) from another author or copyright holder must demonstrate or obtain permission to publish this material under the Creative Commons Attribution 4.0 International (CC BY 4.0) License used by PLOS journals. Please closely review the details of PLOS’s copyright requirements here: PLOS Licenses and Copyright. If you need to request permissions from a copyright holder, you may use PLOS's Copyright Content Permission form.

Please respond directly to this email and provide any known details concerning your material's license terms and permissions required for reuse, even if you have not yet obtained copyright permissions or are unsure of your material's copyright compatibility. Once you have responded and addressed all other outstanding technical requirements, you may resubmit your manuscript within Editorial Manager.

Potential Copyright Issues:

Figure [1]: please (a) provide a direct link to the base layer of the map (i.e., the country or region border shape) and ensure this is also included in the figure legend; and (b) provide a link to the terms of use / license information for the base layer image or shapefile. We cannot publish proprietary or copyrighted maps (e.g. Google Maps, Mapquest) and the terms of use for your map base layer must be compatible with our CC BY 4.0 license.

Note: if you created the map in a software program like R or ArcGIS, please locate and indicate the source of the basemap shapefile onto which data has been plotted.

If your map was obtained from a copyrighted source please amend the figure so that the base map used is from an openly available source. Alternatively, please provide explicit written permission from the copyright holder granting you the right to publish the material under our CC BY 4.0 license.

If you are unsure whether you can use a map or not, please do reach out and we will be able to help you. The following websites are good examples of where you can source open access or public domain maps:

* U.S. Geological Survey (USGS) - All maps are in the public domain. (http://www.usgs.gov)

* PlaniGlobe - All maps are published under a Creative Commons license so please cite “PlaniGlobe, http://www.planiglobe.com, CC BY 2.0” in the image credit after the caption. (http://www.planiglobe.com/?lang=enl)

* Natural Earth - All maps are public domain. (http://www.naturalearthdata.com/about/terms-of-use/).

Note: If the reviewer comments include a recommendation to cite specific previously published works, please review and evaluate these publications to determine whether they are relevant and should be cited. There is no requirement to cite these works unless the editor has indicated otherwise.

**Reviewers' Comments:**

Reviewer's Responses to Questions

**Key Review Criteria Required for Acceptance?**

As you describe the new analyses required for acceptance, please consider the following:

**Methods**

-Are the objectives of the study clearly articulated with a clear testable hypothesis stated?

-Is the study design appropriate to address the stated objectives?

-Is the population clearly described and appropriate for the hypothesis being tested?

-Is the sample size sufficient to ensure adequate power to address the hypothesis being tested?

-Were correct statistical analysis used to support conclusions?

-Are there concerns about ethical or regulatory requirements being met?

Reviewer #1: Methods are mostly clear. It is essential that the authors provide complete qMIQE details for all qPCR assays reported in the manuscript. qMIQE guidelines can be found here: https://pubmed.ncbi.nlm.nih.gov/19246619/. While details for the protozoan assays are mostly included, this information is missing for bacterial pathogens. Also, the authors do not describe a clear justification or rationale for why the specific pathogens they measured were selected (and not others).

Reviewer #2: This cross-sectional study investigated the prevalence of intestinal parasitic and bacterial co-infections among 242 children under 15 years of age from three rural communities in northwestern Madagascar, using light microscopy and real-time PCR. The study found that nearly two thirds of children harbored at least one intestinal pathogen, with Giardia intestinalis and Campylobacter spp. predominating, and that co-infections were common and largely asymptomatic. The authors additionally examined associations between infection patterns and environmental exposures including water source, sanitation access, and livestock contact, and compared haematological parameters across infection groups.

The study objectives are partially articulated. The aim stated at lines 99–103 identifies prevalence estimation, asymptomatic carriage, co-infections, and environmental associations as targets, but no formal testable hypothesis is stated. The inclusion of haematological parameters in the objectives appears as an afterthought with no hypothesis attached.

A community-based cross-sectional design is appropriate for estimating prevalence and describing co-infection patterns in a defined paediatric population. However, the design is not adequate to support causal or directional claims about risk factors, which the authors make in both the results and discussion.

The study population is inadequately described at the point where results are presented. No summary table of participant characteristics is provided before the infection data, making it impossible for the reader to interpret the findings in context. Age distribution is mentioned in passing in the results narrative rather than presented systematically. Sex, site distribution, water source, sanitation access, livestock contact, and antibiotic or deworming exposure are scattered across the text rather than consolidated.

No sample size calculation is provided. The study enrolled 242 children, described implicitly as a convenience sample, with no justification of statistical power for any of the stated objectives. This is a significant gap. For the logistic regression analyses in Tables 5 and 6, several exposure subgroups are critically small, particularly the improved water source group (n=28), rendering regression estimates unstable and the associated confidence intervals unreliable. The authors must either provide a post hoc power calculation or explicitly acknowledge that the study is underpowered for analytical objectives and limit conclusions accordingly.

Fisher's exact test and chi-square tests are applied appropriately for categorical comparisons, and the Kruskal-Wallis test is correctly used for non-parametric haematological data. However, no correction for multiple comparisons is applied despite the large number of statistical tests performed across infection categories, environmental variables, and haematological parameters, raising the risk of type I error throughout. The logistic regression for infection complexity is limited by quasi-complete separation for sanitation variables, which the authors note but do not adequately address. No false discovery rate adjustment is reported. The heading "Statistical Analysis of Infection Prevalence and Risk Factors" appears within the results section at line 401, which is a structural error. The Pearson chi-square value reported at line 343 (X² = 341.68, df = 16) is extremely large relative to the sample size of 242.

Lines 100-103: rationale to include haematological parameters not stated. Why were CBC parameters hypothesized to differ by infection type in an asymptomatic community cohort? Provide justification with a biological rationale and supported by prior literature before data collection.

Line 107: states all samples were collected in August 2025. Were all 242 samples truly collected within a single month? If so, this must be explicitly stated and its implications for representativeness discussed, particularly given seasonal variation in infection patterns.

Lines 125-126: both symptomatic and asymptomatic children were included regardless of recent antimicrobial use. were the included samples diarrheal or non-diarrheal? The bacterial panel used is validated for diarrheal stool. The implications of applying it to non-diarrheal samples must be addressed.

Line 220: Ct cutoff of 38 is used- reference justifying this threshold for each pathogen and this sample matrix (filter paper eluate) is required.

No sample size calculation is presented in methods. how 242 was determined, or explicitly acknowledge convenience sampling as a limitation with its impact on power.

Lines 220-223: Only a single PCR reaction was performed per sample for the in-house protozoan assays, with no internal inhibition control. This is a methodological limitation that fundamentally affects the reliability of negative results. The authors acknowledge this briefly in limitations but do not adequately discuss how it affects interpretation of prevalence estimates throughout the paper.

**********

**Results**

-Does the analysis presented match the analysis plan?

-Are the results clearly and completely presented?

-Are the figures (Tables, Images) of sufficient quality for clarity?

Reviewer #1: No attempt is made to provide quantitative estimates of targets, which is a major limitation of the paper. Given the use of qPCR, these results should be available, and the relative concentrations of targets could help with interpretation of their potential clinical relevance, as explained in this paper: https://www.thelancet.com/journals/lancet/article/PIIS0140-6736(16)31529-X/abstract.

Reviewer #2: The results section requires restructuring. It should begin with a table summarizing participant characteristics (Table 1 equivalent), including number of children under 5 and above, sex distribution, site, water source type, toilet access, livestock contact, and sanitation access stratified by relevant groupings. Without this, the infection data that follows lacks interpretive context. The reader cannot assess the representativeness or risk profile of the study population.

"Improved sanitation" used in Fig 6a legend and throughout requires an operational definition.

Table 1 (primers): Rename appropriately so that the participant characteristics table becomes Table 1. Restructure so that primers are moved to supplementary material or renumbered accordingly.

Tables 2 and 3 (frequency of infections): Given the small sample size, the level of sub-stratification in these tables is not statistically supportable. Many cells contain n=1 or n=2. These tables should be moved to supplementary material. A condensed summary table in the main text is sufficient.

Fig 2 legend, line 305: Typo "od" should be "of."

Fig 5 legend: Typo "symptomps" should be "symptoms."

Line 346: "Higher than expected" requires clarification. Expected under what model or assumption? State explicitly whether this refers to the independence model or a clinical expectation and provide the statistical basis.

Line 349: Children who recently received antibiotics were included in the analysis of bacterial infections. If antibiotic exposure within 28 days preceded sample collection, bacterial detection rates are not interpretable. Were these children excluded from bacterial prevalence analysis?

Line 362: "Multiplathogen" is a typo. Correct to "Multi-pathogen." Also define "multipathogen infection" at first use with a clear operational definition (e.g., detection of two or more distinct pathogen species by any method).

Line 401 heading: "Statistical Analysis of Infection Prevalence and Risk Factors" is a results heading, not a methods heading. This section should be integrated into results.

Table 4: This table is scientifically weak as presented. No sig differences, and infection subgroups include cells with very small numbers (e.g., multiple bacteria n=4). The age range of participants spans infants to adolescents, a group in which CBC reference ranges differ substantially. WBC, lymphocyte counts, and platelet values are not comparable across such a wide age range without age-stratified analysis or adjustment. There is no a priori biological justification for expecting CBC parameters to differ by infection type in an asymptomatic cohort. The authors must either justify the analysis with a clear hypothesis and age-adjusted approach.

No anthropometric measurements (height, weight, MUAC, or stunting status) are presented. In a paediatric cohort in a food-insecure region where environmental enteric dysfunction is a stated concern, this is a significant omission. Were these data collected? If so, they should be included and analyzed in relation to infection status.

Table 5: The take-home message is unclear. The table shows no significant associations for any variable with overall infection status, yet the discussion draws conclusions about environmental risk factors. The authors must reconcile this null result with their interpretive claims and explain what Table 5 contributes to the paper.

Table 6: The inference drawn from this table is limited by the very small number of children using improved water sources (n=28, 11.6%). An adjusted OR of 0.19 from a subgroup of 28 individuals should be interpreted with extreme caution. This limitation must be prominently acknowledged. Additionally, the number of children in mono-infection versus co-infection groups for each exposure variable must be stated in the table or footnote to allow the reader to assess the reliability of the estimates.

**********

**Conclusions**

-Are the conclusions supported by the data presented?

-Are the limitations of analysis clearly described?

-Do the authors discuss how these data can be helpful to advance our understanding of the topic under study?

-Is public health relevance addressed?

Reviewer #1: The conclusions are reasonable and well justified

Reviewer #2: Discussion: Lines 442 onwards: The discussion of environmental risk factors does not address weather or seasonality, despite the fact that all sampling occurred during the dry season. Infection patterns for enteric pathogens, particularly Giardia, Campylobacter, and EIEC, are known to vary by season. The absence of wet season data is a significant limitation that must be discussed here explicitly.

Line 480: The statement that a positive PCR result should be interpreted as an indicator of environmental exposure rather than disease is scientifically problematic as written. PCR positivity in an asymptomatic child does not negate clinical relevance. The authors must clarify: are they suggesting these children should not be treated? If PCR is positioned purely as an environmental indicator, the clinical management implications for individual children must be addressed directly. The framing as written seems misleading.

Line 495: The findings described as novel here (environmental risk factors driving infection, unimproved water associated with parasitic infections, livestock contact associated with bacterial carriage) are well-established including from Madagascar specifically. The authors must demonstrate explicitly how their findings extend or challenge current knowledge rather than restate what is already known.

Line 504: The discussion references school-aged children and behavioral exposure patterns related to school attendance. Were study participants attending school? If so, school attendance and hygiene practices at school should be addressed as a potential exposure and a potential intervention point. If this information was not collected, that is a limitation.

Line 516: The discussion of irrational antibiotic use and VTEC management references a field study finding but does not connect this to the authors' own data. The authors found one child treated with amoxicillin who was positive for VTEC. This is the relevant result to discuss here. The paragraph must be grounded in the authors' findings, not external observations alone.

Line 530: The claim that high subclinical carriage rates demonstrate epidemiological risk independent of symptoms is not new and is not uniquely supported by this dataset. The authors must articulate what this study adds to what is already established by Collard et al. 2022 and Habib et al. 2021, both of which are cited and report very similar findings from Madagascar.

No false discovery rate correction or other adjustment for multiple comparisons is reported despite numerous statistical tests being performed across infection categories, environmental variables, and haematological parameters. This must be addressed in the methods and results.

**********

**Editorial and Data Presentation Modifications?**

Use this section for editorial suggestions as well as relatively minor modifications of existing data that would enhance clarity. If the only modifications needed are minor and/or editorial, you may wish to recommend “Minor Revision” or “Accept”.

Reviewer #1: (No Response)

Reviewer #2: (No Response)

**********

**Summary and General Comments**

Use this section to provide overall comments, discuss strengths/weaknesses of the study, novelty, significance, general execution and scholarship. You may also include additional comments for the author, including concerns about dual publication, research ethics, or publication ethics. If requesting major revision, please articulate the new experiments that are needed.

Reviewer #1: This paper is a well-written, basic analysis of enteric pathogens in children in an understudied context. Although its central insights are not new - high prevalence of enteric pathogen detection in stool from asymptomatic children in high-burden settings is the norm and this has been known for years - it is a useful study that may be of general interest.

Reviewer #2: The limitations section is incomplete. The following must be added:

• wide age range and its effect on CBC interpretation.

• absence of a wet season comparison.

• very small improved-water subgroup limiting the validity of regression estimates.

• inclusion of antibiotic-exposed children in bacterial prevalence analysis.

• inability to distinguish active infection from residual DNA in asymptomatic PCR-positive individuals.

Given the methodological limitations acknowledged by the authors themselves (single stool sample, single PCR reaction, no internal inhibition control for protozoan assays, convenience sample with no formal sample size calculation, extremely small subgroups for regression), the strength of the conclusions requires moderation throughout. The take-home message must be clearly stated: what does this study definitively add to the literature on intestinal co-infections in rural Madagascar that was not previously known?

**********

PLOS authors have the option to publish the peer review history of their article (what does this mean?). If published, this will include your full peer review and any attached files.

If you choose “no”, your identity will remain anonymous but your review may still be made public.

**Do you want your identity to be public for this peer review?**  For information about this choice, including consent withdrawal, please see our Privacy Policy.

Reviewer #1: No

Reviewer #2: **Yes:**  Zehra Jamil

[NOTE: If reviewer comments were submitted as an attachment file, they will be attached to this email and accessible via the submission site. Please log into your account, locate the manuscript record, and check for the action link "View Attachments". If this link does not appear, there are no attachment files.]

**Figure resubmission:**

-->While revising your submission, we strongly recommend that you use PLOS’s NAAS tool (https://ngplosjournals.pagemajik.ai/artanalysis) to test your figure files. NAAS can convert your figure files to the TIFF file type and meet basic requirements (such as print size, resolution), or provide you with a report on issues that do not meet our requirements and that NAAS cannot fix.

After uploading your figures to PLOS’s NAAS tool - https://ngplosjournals.pagemajik.ai/artanalysis, NAAS will process the files provided and display the results in the "Uploaded Files" section of the page as the processing is complete. If the uploaded figures meet our requirements (or NAAS is able to fix the files to meet our requirements), the figure will be marked as "fixed" above. If NAAS is unable to fix the files, a red "failed" label will appear above. When NAAS has confirmed that the figure files meet our requirements, please download the file via the download option, and include these NAAS processed figure files when submitting your revised manuscript.

**Reproducibility:**

To enhance the reproducibility of your results, we recommend that authors of applicable studies deposit laboratory protocols in protocols.io, where a protocol can be assigned its own identifier (DOI) such that it can be cited independently in the future. Additionally, PLOS ONE offers an option to publish peer-reviewed clinical study protocols. Read more information on sharing protocols at https://plos.org/protocols?utm_medium=editorial-email&utm_source=authorletters&utm_campaign=protocols

# Author comment: Author response to Decision Letter 1

**DOI:** 10.1371/journal.pntd.0014519.r002

8 May 2026

Attachment Submitted filename: RESPONSE TO REVIEWERS.docx

# Peer review: Decision Letter 1

**DOI:** 10.1371/journal.pntd.0014519.r003

**Authors:** Joseph M. Vinetz, Luther A Bartelt

10 Jun 2026

PNTD-D-26-00325R1

Chronic exposure to intestinal parasites and bacterial enteropathogens among children in rural Madagascar: implications for asymptomatic carriage and co-infections

PLOS Neglected Tropical Diseases

Dear Dr. Wilczyńska,

Thank you for submitting your manuscript to PLOS Neglected Tropical Diseases. After careful consideration, we feel that it has merit but does not fully meet PLOS Neglected Tropical Diseases's publication criteria as it currently stands. Therefore, we invite you to submit a revised version of the manuscript that addresses the points raised during the review process.

Please submit your revised manuscript by Aug 09 2026 11:59PM. If you will need more time than this to complete your revisions, please reply to this message or contact the journal office at plosntds@plos.org. When you're ready to submit your revision, log on to https://www.editorialmanager.com/pntd/ and select the 'Submissions Needing Revision' folder to locate your manuscript file.

Please include the following items when submitting your revised manuscript:

* A letter that responds to each point raised by the editor and reviewer(s). You should upload this letter as a separate file labeled 'Response to Reviewers'. This file does not need to include responses to any formatting updates and technical items listed in the 'Journal Requirements' section below.

* A marked-up copy of your manuscript that highlights changes made to the original version. You should upload this as a separate file labeled 'Revised Manuscript with Track Changes'.

* An unmarked version of your revised paper without tracked changes. You should upload this as a separate file labeled 'Manuscript'.

If you would like to make changes to your financial disclosure, competing interests statement, or data availability statement, please make these updates within the submission form at the time of resubmission. Guidelines for resubmitting your figure files are available below the reviewer comments at the end of this letter.

As the corresponding author, your ORCID iD is verified in the submission system and will appear in the published article. PLOS supports the use of ORCID, and we encourage all coauthors to register for an ORCID iD and use it as well. Please encourage your coauthors to verify their ORCID iD within the submission system before final acceptance, as unverified ORCID iDs will not appear in the published article. Only the individual author can complete the verification step; PLOS staff cannot verify ORCID iDs on behalf of authors.

We look forward to receiving your revised manuscript.

Kind regards,

Luther A Bartelt

Academic Editor

PLOS Neglected Tropical Diseases

Joseph Vinetz

Section Editor

PLOS Neglected Tropical Diseases

Shaden Kamhawi

co-Editor-in-Chief

PLOS Neglected Tropical Diseases

orcid.org/0000-0003-4304-636XX

Paul Brindley

co-Editor-in-Chief

PLOS Neglected Tropical Diseases

orcid.org/0000-0003-1765-0002

**Additional Editor Comments (if provided):**

**Reviewers' comments:**

Reviewer's Responses to Questions

**Key Review Criteria Required for Acceptance?**

As you describe the new analyses required for acceptance, please consider the following:

**Methods**

-Are the objectives of the study clearly articulated with a clear testable hypothesis stated?

-Is the study design appropriate to address the stated objectives?

-Is the population clearly described and appropriate for the hypothesis being tested?

-Is the sample size sufficient to ensure adequate power to address the hypothesis being tested?

-Were correct statistical analysis used to support conclusions?

-Are there concerns about ethical or regulatory requirements being met?

Reviewer #1: Thank you for the response and for the revisions to the manuscript; it is much improved. However, apart from the protozoan targets, I do not see the full MIQE details for the assays used. Please refer to the MIQE guidelines: https://academic.oup.com/clinchem/article-abstract/55/4/611/5631762. A table in Supporting Information or an annex would be appropriate. While specifying the target genes is helpful, the actual sequences would also be useful for interpretation. If the specific sequences are unavailable because they are proprietary, that would be useful to note. If there are other studies that have validated the pathogen panel, they could be referenced as well.

Reviewer #2: The authors have addressed the comments in their methodology and across the manuscript.

**********

**Results**

-Does the analysis presented match the analysis plan?

-Are the results clearly and completely presented?

-Are the figures (Tables, Images) of sufficient quality for clarity?

Reviewer #1: (No Response)

Reviewer #2: (No Response)

**********

**Conclusions**

-Are the conclusions supported by the data presented?

-Are the limitations of analysis clearly described?

-Do the authors discuss how these data can be helpful to advance our understanding of the topic under study?

-Is public health relevance addressed?

Reviewer #1: (No Response)

Reviewer #2: (No Response)

**********

**Editorial and Data Presentation Modifications?**

Use this section for editorial suggestions as well as relatively minor modifications of existing data that would enhance clarity. If the only modifications needed are minor and/or editorial, you may wish to recommend “Minor Revision” or “Accept”.

Reviewer #1: (No Response)

Reviewer #2: (No Response)

**********

**Summary and General Comments**

Use this section to provide overall comments, discuss strengths/weaknesses of the study, novelty, significance, general execution and scholarship. You may also include additional comments for the author, including concerns about dual publication, research ethics, or publication ethics. If requesting major revision, please articulate the new experiments that are needed.

Reviewer #1: (No Response)

Reviewer #2: (No Response)

**********

PLOS authors have the option to publish the peer review history of their article (what does this mean?). If published, this will include your full peer review and any attached files.

If you choose “no”, your identity will remain anonymous but your review may still be made public.

**Do you want your identity to be public for this peer review?**  For information about this choice, including consent withdrawal, please see our Privacy Policy.

Reviewer #1: No

Reviewer #2: No

[NOTE: If reviewer comments were submitted as an attachment file, they will be attached to this email and accessible via the submission site. Please log into your account, locate the manuscript record, and check for the action link "View Attachments". If this link does not appear, there are no attachment files.]

**Figure resubmission:**

While revising your submission, we strongly recommend that you use PLOS’s NAAS tool (https://ngplosjournals.pagemajik.ai/artanalysis) to test your figure files. NAAS can convert your figure files to the TIFF file type and meet basic requirements (such as print size, resolution), or provide you with a report on issues that do not meet our requirements and that NAAS cannot fix.

After uploading your figures to PLOS’s NAAS tool - https://ngplosjournals.pagemajik.ai/artanalysis, NAAS will process the files provided and display the results in the "Uploaded Files" section of the page as the processing is complete. If the uploaded figures meet our requirements (or NAAS is able to fix the files to meet our requirements), the figure will be marked as "fixed" above. If NAAS is unable to fix the files, a red "failed" label will appear above. When NAAS has confirmed that the figure files meet our requirements, please download the file via the download option, and include these NAAS processed figure files when submitting your revised manuscript.

**Reproducibility:**

To enhance the reproducibility of your results, we recommend that authors of applicable studies deposit laboratory protocols in protocols.io, where a protocol can be assigned its own identifier (DOI) such that it can be cited independently in the future. Additionally, PLOS ONE offers an option to publish peer-reviewed clinical study protocols. Read more information on sharing protocols at https://plos.org/protocols?utm_medium=editorial-email&utm_source=authorletters&utm_campaign=protocols

# Author comment: Author response to Decision Letter 2

**DOI:** 10.1371/journal.pntd.0014519.r004

11 Jun 2026

Attachment Submitted filename: Response to reviewers (2).docx

# Editor report: Decision Letter 2

**DOI:** 10.1371/journal.pntd.0014519.r005

**Authors:** Joseph M. Vinetz, Luther A Bartelt

30 Jun 2026

Dear phD Wilczyńska,

We are pleased to inform you that your manuscript 'Chronic exposure to intestinal parasites and bacterial enteropathogens among children in rural Madagascar: implications for asymptomatic carriage and co-infections' has been provisionally accepted for publication in PLOS Neglected Tropical Diseases.

Before your manuscript can be formally accepted you will need to complete some formatting changes, which you will receive in a follow up email. A member of our team will be in touch with a set of requests.

Please note that your manuscript will not be scheduled for publication until you have made the required changes, so a swift response is appreciated.

IMPORTANT: The editorial review process is now complete. PLOS will only permit corrections to spelling, formatting or significant scientific errors from this point onwards. Requests for major changes, or any which affect the scientific understanding of your work, will cause delays to the publication date of your manuscript.

Should you, your institution's press office or the journal office choose to press release your paper, you will automatically be opted out of early publication. We ask that you notify us now if you or your institution is planning to press release the article. All press must be co-ordinated with PLOS.

Thank you again for supporting Open Access publishing; we are looking forward to publishing your work in PLOS Neglected Tropical Diseases.

Best regards,

Luther A Bartelt

Academic Editor

PLOS Neglected Tropical Diseases

Joseph Vinetz

Section Editor

PLOS Neglected Tropical Diseases

Shaden Kamhawi

co-Editor-in-Chief

PLOS Neglected Tropical Diseases

orcid.org/0000-0003-4304-636XX

Paul Brindley

co-Editor-in-Chief

PLOS Neglected Tropical Diseases

orcid.org/0000-0003-1765-0002

***********************************************************

p.p1 {margin: 0.0px 0.0px 0.0px 0.0px; line-height: 16.0px; font: 14.0px Arial; color: #323333; -webkit-text-stroke: #323333}span.s1 {font-kerning: none

# Editor report: Acceptance letter

**DOI:** 10.1371/journal.pntd.0014519.r006

**Authors:** Joseph M. Vinetz, Luther A Bartelt

Dear phD Wilczyńska,

We are delighted to inform you that your manuscript, "Chronic exposure to intestinal parasites and bacterial enteropathogens among children in rural Madagascar: implications for asymptomatic carriage and co-infections," has been formally accepted for publication in PLOS Neglected Tropical Diseases.

We have now passed your article onto the PLOS Production Department who will complete the rest of the publication process. All authors will receive a confirmation email upon publication.

The corresponding author will soon be receiving a typeset proof for review, to ensure errors have not been introduced during production. Please review the PDF proof of your manuscript carefully, as this is the last chance to correct any scientific or type-setting errors. Please note that major changes, or those which affect the scientific understanding of the work, will likely cause delays to the publication date of your manuscript. Note: Proofs for Front Matter articles (Editorial, Viewpoint, Symposium, Review, etc...) are generated on a different schedule and may not be made available as quickly.

Soon after your final files are uploaded, the early version of your manuscript will be published online unless you opted out of this process. The date of the early version will be your article's publication date. The final article will be published to the same URL, and all versions of the paper will be accessible to readers.

For Research Articles, you will receive an invoice from PLOS for your publication fee after your manuscript has reached the completed accept phase. If you receive an email requesting payment before acceptance or for any other service, this may be a phishing scheme. Learn how to identify phishing emails and protect your accounts at https://explore.plos.org/phishing.

Thank you again for supporting open-access publishing; we are looking forward to publishing your work in PLOS Neglected Tropical Diseases.

Best regards,

Shaden Kamhawi

co-Editor-in-Chief

PLOS Neglected Tropical Diseases

Paul Brindley

co-Editor-in-Chief

PLOS Neglected Tropical Diseases
